# Supplementary material for: OptiBreech collaborative care versus standard care for women with a breech-presenting fetus at term: A pilot parallel group randomised trial to evaluate the feasibility of a randomised trial nested within a cohort
Source: PLoS One. 2023 Nov 15;18(11):e0294139. doi: 10.1371/journal.pone.0294139 (PMC10650999; doi:10.1371/journal.pone.0294139)
Supplement: S1 Checklist — Combined CONSORT checklist extension for randomised pilot and feasibility trials and GRIPP2 short form for reporting patient and public involvement in health and social care research. (DOCX) [file pone.0294139.s001.docx]

S1. CONSORT / GRIPP2 checklist. Combined CONSORT checklist extension for randomised pilot and feasibility trials[1] and GRIPP2 short form for reporting patient and public involvement in health and social care research(12).

ABSTRACT

| **Section / topic and item number** | **Checklist item** | **Location** | **Explanation** |
| --- | --- | --- | --- |
| Title | Identification of study as randomised pilot or feasibility trial | Abstract | Identified as a pilot randomised trial to evaluate feasibility. |
| Trial design | Description of pilot trial design | Abstract | Parallel group design identified |
| *Methods:* | | | |
| Participants | Eligibility criteria for participants and the settings where the pilot trial was conducted | Abstract | Eligibility criteria, settings and time period identified. |
| Interventions | Interventions intended for each group | Abstract | OptiBreech Care pathway versus standard care identified |
| Objective | Specific objectives of the pilot trial | Abstract | Primary objectives identified. |
| Outcome | Prespecified assessment or measurement to address the pilot trial objectives | Abstract | Primary outcomes and feasibility-tested outcomes identified. |
| Randomisation | How participants were allocated to interventions | Abstract | 1:1 parallel group allocation identified. |
| Blinding (masking) | Where or not participants, caregivers, and those assessing the outcomes were blinded to group assignment | Abstract | Lack of blinding identified. |
| *Results:* | | | |
| Numbers randomised | Number of participants screened and randomised to each group for the pilot objectives | Abstract | Identified |
| Recruitment | Trial status | Abstract | Identified |
| Numbers analysed | Number of participants analysed in each group for the pilot objectives | Abstract | Complete primary outcomes dataset identified |
| Outcome | Results for the pilot objectives, including any expressions of uncertainty | Abstract | Lack of power for feasibility-tested outcomes identified |
| Harms | Important adverse events or side effects | Abstract | Severe neonatal mortality reported. |
| Conclusions | General interpretation of the results of pilot trial and their implications for the future definitive trial | Abstract | Included |
| Trial registration | Registration number for pilot rial and name of trial register | Abstract | Included with design description |
| Funding | Source of funding for pilot trial | Abstract | Included |

GRIPP2 short form

| **Section / topic and item number** | **Checklist item** | **Location** | **Explanation** |
| --- | --- | --- | --- |
| 1: Aim | Report the aim of PPI(E) in the study | Background | Describing the aim and the primary population included in PPIE work. |
| 2: Methods | Provide a clear description of the methods use for PPI(E) in the study | PPIE heading |  |
| 3: Study results | Outcomes – Report the results of PPI(E) in the study, including both positive and negative outcomes | Results | Sub-group analyses reflecting the priorities identified in PPIE work are reported. |
| 4: Discussion and conclusions | Outcomes – Comment on the extent to which PPI(E) influenced the study overall. Describe positive and negative effects | Discussion | Discussion around equity of access issues raised by participants. |
| 5: Reflections / critical perspective | Comment critically on the study, reflecting on the things that went well and those that did not, so others can learn from this experience | Methods / PPIE heading and Discussion |  |

| **Section / topic and item number** | **Checklist item** | **Location** | **Explanation / Modification** |
| --- | --- | --- | --- |
| Title and abstract | | | |
| 1a | Identification as a pilot or feasibility randomised trial in the title | Title | Pilot trial to assess feasibility – both mentioned in title |
| 1b | Structured summary of pilot trial design, methods, results, and conclusions | Abstract | Consort abstract checklist above |
| Introduction | | | |
| *Background and objectives:* | | | |
| 2a | Scientific background and explanation of rationale for future definitive trial, and reasons for randomised pilot trial | Background and Research in Context panel |  |
| 2b | Specific objectives or research questions for pilot trial | Background | Overall aim of determining feasibility explained |
| Methods | | | |
| *Trial design:* | | | |
| 3a | Description of pilot trial design (such as parallel, factorial) including allocation ratio | Methods, study design |  |
| 3b | Important changes to methods after pilot trial commencement (such as eligibility criteria, with reasons) | N/A |  |
| Participants | | | |
| 4a | Eligibility criteria for participants | Participants |  |
| 4b | Settings and locations where the data were collected | Study design |  |
| 4c | How participants were identified and consented | Consent process |  |
| Intervention | | | |
| 5 | The interventions for each group with sufficient details to allow replication, including how and when they were actually administered. | Procedures | A link is provided to the full prospectively registered protocol with the TIDieR checklist. |
| Outcomes | | | |
| 6a | Completely defined prespecified assessments or measurements to address each pilot trial objective specified in 2b, including how and when they were assessed | Outcomes, with further details provided in Results | A link is provided to the full prospectively registered protocol with detailed descriptions of outcome data collected. |
| 6b | Any changes to pilot trial assessments or measurements after the pilot trial commenced, with reasons | Results | Discussion of modification of eCRF to collect data about ECVs not performed as planned. |
| 6c | If applicable, prespecified criteria used to judge whether, or how, to proceed with future definitive trial | Trial feasibility criteria |  |
| Sample size | | | |
| 7a | Rationale for numbers in the pilot trial | Statistical analysis |  |
| 7b | When applicable, explanation of any interim analyses and stopping guidelines | Trial feasibility criteria and Results |  |
| Randomisation | | | |
| 8a | Method used to generate the random allocation sequence | Randomisation and masking |  |
| 8b | Type of randomisation(s); details of any restriction (such as blocking and block size) | Randomisation and masking |  |
| Allocation concealment mechanism | | | |
| 9 | Mechanism used to implement the random allocation sequence (such as sequentially numbered containers), describing any steps taken to conceal the sequence until interventions were assigned | Randomisation and masking |  |
| Implementation of randomisation procedures | | | |
| 10 | Who generated the random allocation sequence, enrolled participants, and assigned participants to interventions | Randomisation and masking |  |
| Blinding | | | |
| 11a | If done, who was blinded after assignment to interventions (eg, participants, care providers, those assessing outcomes) | Randomisation and masking |  |
| 11b | If relevant, description of the similarity of interventions | Procedures | A link is provided to the full prospectively registered protocol with the TIDieR checklist. |
| Analytical methods | | | |
| 12a | Methods used to address each pilot trial objective whether qualitative or quantitative | Statistical analysis |  |
| 12b | Methods for additional analyses, such as subgroup analyses and adjusted analyses | Statistical analysis |  |
| Results | | | |
| Participant flow (a diagram is strongly recommended): | | | |
| 13a | For each group, the numbers of participants who were approached and/or assessed for eligibility, randomly assigned, received intended treatment, and were assessed for each objective | Figure 1 | Our protocol did not specify tracking those who were approached and/or assessed for eligibility. This figure would be affected by women transferring in for care. |
| 13b | For each group, losses and exclusions after randomisation, together with reasons | Figure 1 | Withdrawals shown |
| Recruitment: | | | |
| 14a | Dates defining the periods of recruitment and follow-up | Results, first section |  |
| 14b | Why the pilot trial ended or was stopped | Explained following Table 5 |  |
| Baseline data: | | | |
| 15 | A table showing baseline demographic and clinical characteristics for each group | Table 4 |  |
| Numbers analysed: | | | |
| 16 | For each objective, number of participants (denominator) included in each analysis. If relevant, these numbers should be by randomised group | Tables |  |
| Outcomes and estimation: | | | |
| 17a | For each objective, results including expressions of uncertainty, such as 95% confidence interval) for any estimates. If relevant, these results should be by randomised group | Tables | Expressions of uncertainty included for recruitment rates (primary outcome) and planned VBB (significant outcome at interim analysis and basis for decision to stop randomisation early). |
| 17b | Effect sizes | Tables | As above |
| Ancillary analyses: | | | |
| 18 | Results of any other analyses performed that could be used to inform the future definitive trial | Subgroup analyses performed as planned, Table 7 | Subgroup analysis by ethnic group also reported, Table 8 |
| Harms: | | | |
| 19 | All important harms or unintended effects in each group (for specific guidance see CONSORT for harms) | Table 9 |  |
| 19a | If relevant, other important unintended consequences | Table 9 |  |
| Discussion: | | | |
| Limitations: | | | |
| 20 | Pilot trial limitations, addressing sources of potential bias and remaining uncertainty about feasibility | Inability to blind participants and professionals discussed, not all women randomised as planned |  |
| Generalisability | | | |
| 21 | Generalisability (applicability) of pilot trial methods and findings to future definitive trial and other studies | Discussed why 1:1 randomisation is not possible for this intervention |  |
| Interpretation: | | | |
| 22 | Interpretation consistent with pilot trial objectives and findings, balancing potential benefits and harms, and considering other relevant evidence | Similarity of recruitment rates to OptiBreech 1 discussed |  |
| 22a | Implications for progression from pilot to future definitive trial, including any proposed amendments | Discussed need for cluster randomisation and blinding in statistical analysis |  |
| Other information: | | | |
| Registration: | | | |
| 23 | Registration number for pilot trial and name of trial registry | Study design |  |
| Protocol: | | | |
| 24 | Where the pilot trial protocol can be accessed, if available | Procedures, References |  |
| Funding: | | | |
| 25 | Sources of funding and other support (such as supply of drugs), role of funders | Acknowledgements |  |
| 26 | Ethical approval or approval by research review committee, confirmed with reference number | Study Design |  |

1. Eldridge SM, Chan CL, Campbell MJ, Bond CM, Hopewell S, Thabane L, et al. CONSORT 2010 statement: Extension to randomised pilot and feasibility trials. Pilot Feasibility Stud. 2016;2: 1–32. doi:10.1186/S40814-016-0105-8/FIGURES/2

2. Staniszewska S, Brett J, Simera I, Seers K, Mockford C, Goodlad S, et al. GRIPP2 reporting checklists: tools to improve reporting of patient and public involvement in research. BMJ. 2017;358: 3453. doi:10.1136/BMJ.J3453
