# Supplementary figures and images for: OptiBreech collaborative care versus standard care for women with a breech-presenting fetus at term: A pilot parallel group randomised trial to evaluate the feasibility of a randomised trial nested within a cohort
Source: PLoS One. 2023 Nov 15;18(11):e0294139. doi: 10.1371/journal.pone.0294139 (PMC10650999; doi:10.1371/journal.pone.0294139)

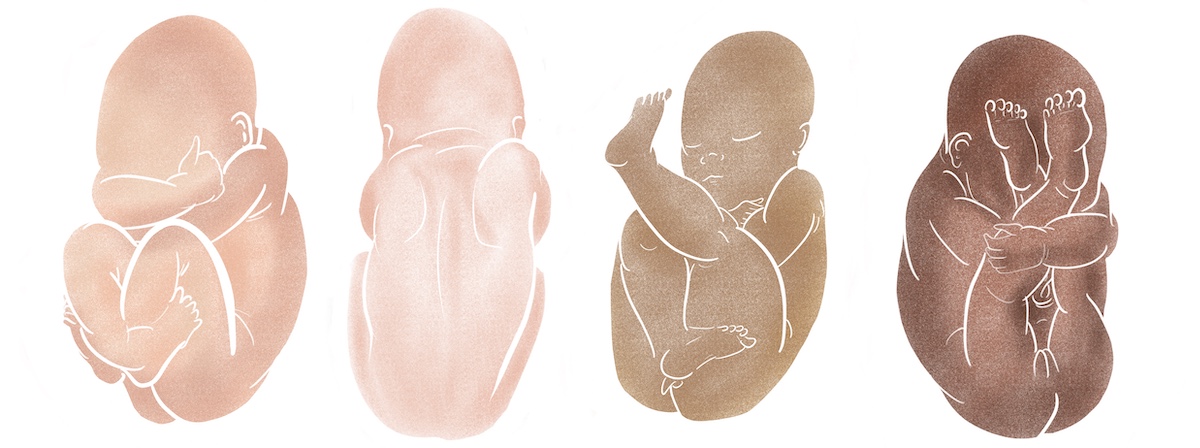

Supplement: S1 Fig — (JPEG) [file pone.0294139.s002.jpeg]
