# Supplementary material for: OptiBreech collaborative care versus standard care for women with a breech-presenting fetus at term: A pilot parallel group randomised trial to evaluate the feasibility of a randomised trial nested within a cohort
Source: PLoS One. 2023 Nov 15;18(11):e0294139. doi: 10.1371/journal.pone.0294139 (PMC10650999; doi:10.1371/journal.pone.0294139)
Supplement: S1 File — (DOCX) [file pone.0294139.s003.docx]

# Protocol details

## Title

The OptiBreech Care Trial: a feasibility study for a pragmatic trial of care for women with a breech-presenting baby at term

## Contact details

**Sponsor**

Name of Sponsoring Organisation: King’s College London

Name of Sponsor Representative: Professor Reza Razavi

Address: Vice President & Vice Principal (Research)

Room 5.31, James Clerk Maxwell Building, 57 Waterloo Road, London SE1 8WA

Telephone: +44 (0)207 848 3224

E-mail: Reza.Razavi@kcl.ac.uk

**Chief Investigator**

Name: Dr Shawn Walker, NIHR Advanced Fellow

Address: King’s College London Department of Women and Children’s Health, 10^th^ Floor North Wing, St Thomas’ Hospital, Westminster Bridge Rd, London, SE1 7EH

Telephone: 07947819122

E-mail: Shawn.Walker@kcl.ac.uk

**Research Assistant:**

Name: Tisha Dasgupta, MSc Public Health

E-mail: Tisha.Dasgupta@kcl.ac.uk

**Health Economist**: TBA

**Service User Co-Investigators**: Sharna Reid and Sarah Hunter

**PPI Lead:** Laura Berkeley (service user)

## Additional members of the research team

Name: Professor Andrew Shennan (Co-I)

E-mail: Andrew.Shennan@kcl.ac.uk

Name: Professor Jane Sandall (Co-I)

E-mail: Jane.Sandall@kcl.ac.uk

Statistical Guidance: Dr Kirsty Logan (Co-I)

E-mail: kirsty.logan@kcl.ac.uk

Health Economics Guidance: Professor Julia Fox-Rushby (Co-I)

E-mail: Julia.Fox-Rushby@kcl.ac.uk

## Participating Sites and Principal Investigators

**Lead Site:** Chelsea and Westminster Hospitals NHS Trust (Chelsea & Westminster site)

Principal Investigator: Dr Shawn Walker

E-mail: Shawn.Walker@kcl.ac.uk

**Site 2:** Chelsea and Westminster Hospitals NHS Trust (West Middlesex site)

Principal Investigator: Lauren Trepte

E-mail: lauren.trepte2@nhs.net

**Site 3:** Kingston Hospital NHS Foundation Trust

Principal Investigator: Emma Spillane

E-mail: E.Spillane@nhs.net

**Site 4:** Surrey and Sussex Healthcare NHS Trust

Principal Investigator: Kate Stringer

E-mail: Kate.Stringer@nhs.net

## Protocol details

Version number 1.0, 7-SEP-21

# Contents

[1 Protocol details 1](#_Toc86663150)

[Title 1](#_Toc86663151)

[Contact details 1](#_Toc86663152)

[Additional members of the research team 2](#_Toc86663153)

[Participating Sites and Principal Investigators 2](#_Toc86663154)

[Protocol details 3](#_Toc86663155)

[Contents 3](#_Toc86663156)

[Tables and Figures 7](#_Toc86663157)

[2 Abbreviations 7](#_Toc86663158)

[3 Summary 9](#_Toc86663159)

[4 Introduction 10](#_Toc86663160)

[What is the problem being addressed? 10](#_Toc86663161)

[Why is this research important in terms of improving health and/or wellbeing of the public and/or to patients and health care services? 11](#_Toc86663162)

[Review of existing evidence 12](#_Toc86663163)

[Feasibility Testing of the OptiBreech model of care so far 15](#_Toc86663164)

[Patient and public involvement (PPI) 18](#_Toc86663165)

[5 Aim, objectives and outcomes 20](#_Toc86663166)

[Objectives 20](#_Toc86663167)

[Primary Outcomes 21](#_Toc86663168)

[Secondary Outcomes 21](#_Toc86663169)

[6 Study design and Flowchart 22](#_Toc86663170)

[The OptiBreech Multiple trial cohort (OptiBreech mTC) 22](#_Toc86663171)

[The OptiBreech-ECV Trial 23](#_Toc86663172)

[Flowchart 24](#_Toc86663173)

[Research Timeline 25](#_Toc86663174)

[7 Participant Selection 25](#_Toc86663175)

[Sites 25](#_Toc86663176)

[Participants 26](#_Toc86663177)

[OptiBreech mTC – Cohort inclusion criteria 26](#_Toc86663178)

[Cohort Exclusion criteria 26](#_Toc86663179)

[OptiBreech-ECV Trial – Additional inclusion criteria for randomisation 27](#_Toc86663180)

[Trial exclusion criteria 27](#_Toc86663181)

[8 Study procedures 27](#_Toc86663182)

[Participant recruitment 27](#_Toc86663183)

[Breech presentation diagnosed in Labour 30](#_Toc86663184)

[Screening Procedures 31](#_Toc86663185)

[Randomisation Procedures 31](#_Toc86663186)

[Masking and other measures taken to avoid bias 32](#_Toc86663187)

[Masking 32](#_Toc86663188)

[Schedule of treatment for each visit 33](#_Toc86663189)

[Follow-up Procedures 33](#_Toc86663190)

[Radiology assessments 34](#_Toc86663191)

[End of Study Definition 34](#_Toc86663192)

[9 Description of intervention 34](#_Toc86663193)

[Tidier Checklist 35](#_Toc86663194)

[Logic model 39](#_Toc86663195)

[Proficiency criteria 39](#_Toc86663196)

[Counselling 40](#_Toc86663197)

[Autonomy and individualised care 41](#_Toc86663198)

[Attendance in labour and clinical responsibility 42](#_Toc86663199)

[Maternal birthing posture 43](#_Toc86663200)

[Use of algorithm 43](#_Toc86663201)

[Recordkeeping 44](#_Toc86663202)

[10 Assessment of safety 44](#_Toc86663203)

[Ethics Reporting 44](#_Toc86663204)

[Trial Steering Committee 47](#_Toc86663205)

[Ethics and regulatory approvals 47](#_Toc86663206)

[11 Compliance and withdrawal 47](#_Toc86663207)

[Subject compliance 47](#_Toc86663208)

[Dropout of participants 47](#_Toc86663209)

[Protocol compliance 48](#_Toc86663210)

[12 Data 49](#_Toc86663211)

[Data to be collected 49](#_Toc86663212)

[Data quality and validity 54](#_Toc86663213)

[Data handling and record keeping 55](#_Toc86663214)

[Anonymisation of data 56](#_Toc86663215)

[13 Statistical considerations 56](#_Toc86663216)

[Sample size calculation 56](#_Toc86663217)

[Statistical analysis 57](#_Toc86663218)

[14.3 Interim analysis and data monitoring 59](#_Toc86663219)

[Green/Amber/Red Criteria for recommending a full RCT 59](#_Toc86663220)

[Monitoring, quality control and assurance 60](#_Toc86663221)

[14 Ethical considerations 60](#_Toc86663222)

[COVID-19 precautions 60](#_Toc86663223)

[Peer and Ethical Review 61](#_Toc86663224)

[Distress and concerns 61](#_Toc86663225)

[PPI & Ethics 63](#_Toc86663226)

[Accessibility 63](#_Toc86663227)

[15 Study Oversight Arrangements 64](#_Toc86663228)

[Trial Management Group (TMG) 64](#_Toc86663229)

[Trial Steering Committee (TSC) 64](#_Toc86663230)

[Meeting schedule 65](#_Toc86663231)

[16 Financing and insurance 65](#_Toc86663232)

[Financing 65](#_Toc86663233)

[Insurance 65](#_Toc86663234)

[17 Reporting and Dissemination 66](#_Toc86663235)

[Transparency and openness strategy 66](#_Toc86663236)

[Authorship Policy 66](#_Toc86663237)

[Publications 67](#_Toc86663238)

[18 References 68](#_Toc86663239)

[19 Definitions 74](#_Toc86663240)

[20 Appendix 1: OptiBreech Practice Guideline 75](#_Toc86663241)

[Background 75](#_Toc86663242)

[Definition of proficiency 75](#_Toc86663243)

[Commence breech pathway 76](#_Toc86663244)

[Biometric Growth Ultrasound Scans 77](#_Toc86663245)

[Labour Care 77](#_Toc86663246)

[Monitoring 77](#_Toc86663247)

[Progress in second stage of labour 78](#_Toc86663248)

[Neonatal Care 79](#_Toc86663249)

[Special Clinical Situations 79](#_Toc86663250)

[Care of non-extended breech presentation 79](#_Toc86663251)

[Fetal size 80](#_Toc86663252)

[Prior Caesarean Section 80](#_Toc86663253)

[Assessment of Fidelity 81](#_Toc86663254)

# Tables and Figures

[Figure 1: Study Design Flowchart 24](#_Toc86663255)

[Figure 2: Research Timeline 25](#_Toc86663256)

[Figure 3: Logic Model for OptiBreech Care 39](#_Toc86663257)

[Figure 4: Flow of women/birthing people through the breech care pathway 57](#_Toc86663258)

[Table 1: Results of Physiological Breech Birth training 15](#_Toc86663259)

[Table 2: Results of OptiBreech 1 16](#_Toc86663260)

[Table 3: TiDIER Checklist: Comparison of standard care (‘offer ECV’) with the Intervention (‘offer OptiBreech vaginal breech birth care’) 35](#_Toc86663261)

[Table 4: Information with regards to Safety Reporting 45](#_Toc86663262)

# Abbreviations

AE: adverse events

AMU: alongside midwifery unit

CI: chief investigator, confidence interval

Co-I: co-investigator

CRF: case report form

DMC: data monitoring committee

FMU: free-standing midwifery unit

GCP: good clinical practice

HCP: health care professional

HRA: health research authority

ITS: interrupted time series

KCL: King's college London

NICE: National Institute for Health and Care Excellence

NNU: neonatal unit

OOH: out of hospital

OU: obstetric unit

PBB: physiological breech birth

PI: principal Investigator

PIS: participant information sheet

PPI: patient and public involvement

RA: research assistant

RCM: Royal College of Midwives

RCOG: Royal College of Obstetricians and Gynaecologists

RDS: Research Design Service

REC: research ethics committee

R&D: research and development authority

SAE: serious adverse event

SOP: standard operating procedure

TMG: project management group

TSC: trial steering committee

TWiC: trial within a cohort

VBB: vaginal breech birth

UK: United Kingdom

# Summary

| Scientific Title |  | The OptiBreech Care Trial: a feasibility study for a pragmatic trial of care for women with a breech-presenting baby at term |
| --- | --- | --- |
| Protocol Short Title/Acronym |  | OptiBreech Care |
| Protocol Version number and Date |  | V1.1 1 Nov 2021 |
| IRAS Number |  | 303028 |
| CPMS |  | 50898 |
| ISRCTN Reference |  | ISRCTN14521381 |
| REC Reference |  | 21/LO/0808 |
| Study Duration |  | 1 December 2021 – 31 August 2023  Randomisation planned for December 2021 – May 2022 |
| Methodology |  | Pilot trial within a feasibility study |
| Sponsor name |  | King’s College London |
| Chief Investigator |  | Dr Shawn Walker |
| Funder Name |  | National Institute for Health Research (NIHR) |
| Funder Reference |  | NIHR300582 |
| Purpose of study |  | Determine the feasibility of conducting a Trial within Cohort of planned vaginal birth with OptiBreech care versus planned external cephalic version |
| Primary objective |  | Identify how many women will consent to randomisation and accept the care pathway to which they are allocated, to inform estimations for a full RCT. |
| Secondary objective (s) |  | 1. Measure the completeness of outcome data and time required to gather it 2. Identify the relevant resources and health services used and test appropriate methods for their measurement 3. Describe preliminary safety outcomes for the cohort 4. Determine with a Trial Steering Committee (TSC) whether a trial is feasible and offers value for a future policy change |
| Number of Subjects/Patients |  | Total: 154 women  50 women recruited to the cohort observational study without randomisation (estimate)  104 women randomised in an internal pilot trial of OptiBreech care versus ECV |
| Study Design |  | Pilot trial |
| Outcomes |  | Primary outcomes: recruitment and retention rates, accuracy and completion of data set, fidelity to intervention  Secondary outcomes: outcomes as a result of the intervention whose feasibility we are testing, including safety outcomes |
| Main Inclusion Criteria |  | **Cohort:**   - Breech presentation >32 weeks of pregnancy, referred for specialist care related to breech presentation - Breech presentation >37 weeks of pregnancy discovered in labour - Requesting or preferring a vaginal birth, with no absolute contraindication   **Trial within cohort:**   - no relative contraindication associated with higher risk of vaginal breech birth or ECV - no indication for induction prior to 41 weeks at the time of recruitment |
| Statistical Methodology and Analysis |  | Descriptive statistics will be used to report recruitment and adherence rates. The feasibility study is not powered to detect a difference in safety, but at its conclusion, all SAEs and safety outcome measures will be reviewed by the Trial Steering Committee. |

# Introduction

## What is the problem being addressed?

A lack of high-quality, recent evidence undermines shared decision-making for women with breech pregnancies. Across the UK and internationally, women have raised concerns about a lack of support.^1–6^ While some women are relieved to be offered a caesarean section (CS), other women report no option but to deliver by CS, causing ‘stress, anger, fear and injustice,'^7^ and in some cases long-term emotional trauma.^8^ Some feel pressured to attempt an external cephalic version (ECV).^9–11^ An ECV is a procedure to manually turn the fetus head-down using pressure on the maternal abdomen.^12^ Some experience the procedure as very painful, with over 10% describing it as ‘intolerable.’^13^

Some providers discourage breech births due to a lack of confidence arising from minimal experience^14^ and evidence that CS reduces the risk of perinatal mortality and severe morbidity compared to classical/supine methods of breech delivery (RR 0.07, 95% CI 0.02 to 0.29, one study, 1025 women).^15^ This is understandable but out of line with individualised decision-making.^16^ Two year outcomes show no differences in ‘death or neurodevelopmental delay’ (RR 1.09, 95% CI 0.52 to 2.30, one study, 920 children), and more infants who had been allocated to planned CS delivery had medical problems at two years (RR 1.41, 95% CI 1.05 to 1.89, one study, 843 children).^15^ Supporting the choice of breech birth may reduce risks in future pregnancies for both mothers and babies, such as morbidly adherent placentas and elevated levels of stillbirths.^15,17^ Facilitating planned vaginal births for women who choose them also enables younger obstetricians and midwives to learn breech skills, potentially improving the safety of unexpected breech births.

Breech presentation occurs in 4% (1:25) of term pregnancies.^16^ As many as 35-58% of women may prefer to plan a breech birth, but this is highly dependent on the type of counselling they receive.^18,19^ Yet a 2014 survey of UK maternity units found that only 27% offered support for a vaginal breech birth.^20^ Some hospitals have created breech clinics and/or an on-call team to revive breech skills;^21,22^ these attract women who lack local support.^23,24^ In some hospitals, the vaginal breech birth rate can be as high as 6-11% of the total birth rate due to women travelling to experienced providers,^23,24^ compared to 0.4% of the total birth rate in the UK.^25^ This suggests inequity and demand for skilled breech birth care.

## Why is this research important in terms of improving health and/or wellbeing of the public and/or to patients and health care services?

PPI work to develop this proposal confirmed research indicating women in the UK are experiencing a lack of appropriate support. Our very first public call for PPI participants in 2019 resulted in three currently pregnant women reaching out for support, claiming they had received biased counselling with ‘no statistics,’ and their providers were unable to facilitate a vaginal breech birth. We have received a steady stream of requests for support since then. Women know they are entitled to accurate information and choice, and they know this is not good enough.

Over 96% of all term breech babies are born by CS in the UK,^25^ and breech is the indication for 14% of all CS in countries with a low perinatal mortality rate.^26^ The majority of breech presentations occur in first pregnancies, contributing significantly to the most common indication for surgical delivery: previous CS.^26^ Elective CS offers some benefits for babies but also creates risks for mothers, especially those who plan to have further children.^27^ Rising rates of morbidly adherent placenta, resulting from previous uterine scars, were highlighted in the last confidential enquiry into maternal deaths and morbidity.^28^ Term caesarean breech delivery in the first pregnancy has been associated with increased risk for maternal and neonatal morbidity in subsequent pregnancies in two national cohort studies, based in the Netherlands^29^ and Finland.^30^ High rates of CS will also have economic effects on the health service, although these are unknown in the context of breech pregnancies – a gap this work aims to address.

Up to 30% of breech presentations are also first discovered in labour,^31^ when the maternal risks associated with emergency CS are higher. For example, a CS performed at full dilatation carries eight times more risk of maternal death than one performed earlier (OR 7.96 95%CI 1.61-39.39).^32^ Additionally, CS performed late in labour increases the risk of subsequent preterm birth six fold.^33^ A loss of breech skills over the last few decades has introduced additional maternal and neonatal risks for these unexpected breech births.^34^ Despite representing only 0.4% of all births,^25^ vaginal breech births accounted for 12% of NHS litigation costs related to cerebral palsy in a recent review.^35^ All but one of these were unexpected, with the breech presentation detected for the first time late in labour.

The RCOG summary of evidence suggests that with skilled and experienced practitioners, breech birth may be ‘nearly as safe as cephalic birth’ (perinatal mortality per 1000: CS=0.5, cephalic birth=1, breech birth=2).^16^  To reduce the CS rate for breech, most women whose babies present breech at term are recommended an ECV.^36^ But ECV has not been shown to improve outcomes for babies, compared to no ECV, in multiple Cochrane Reviews.^37^

## Review of existing evidence

The Cochrane Review on ‘Planned caesarean section for term breech delivery’ reports short-term benefits for infants with no short-term differences for mothers and no long-term differences for children when planned CS is compared to planned breech birth (risk ratios outlined above).^15^ The review includes three trials. Each of these were conducted prior to 2000, none of them include physiological breech birth (PBB) methods,^38^ and none of them includes a care pathway intervention specifically designed to improve the safety of vaginal breech birth. The review concludes by recommending research on strategies to improve the safety of vaginal breech delivery. In a secondary analysis of the largest trial, the presence of an experienced midwife or obstetrician was the only intervention demonstrated to lower risks associated with vaginal breech birth (OR: 0.30, 95% CI, 0.13-0.68, *p*=.004).^39^

The Cochrane Review on ‘External cephalic version for breech presentation at term’ reports benefits for mothers with no significant differences for infants when ECV is compared with no ECV.^37^ In this review, ECV reduces the CS rate compared to no ECV (RR 0.57, 95% CI 0.40 to 0.82, evidence graded very low). The review found no significant differences in neonatal outcomes, including the incidence of Apgar score ratings below seven at one minute or five minutes, low umbilical vein pH levels, neonatal admission, and perinatal death.

In a large UK cohort study, ECV was associated with a combined stillbirth and neonatal mortality rate of 1.9 per 1000,^40^ including all subsequent modes of delivery: cephalic births, breech births and CS. A population-level cohort study in the Netherlands associated planned vaginal breech birth with a perinatal mortality rate of 1.6 per 1000, and 1.3 per 1000 when cases undiagnosed before labour were excluded.^41^ Also in the Netherlands during the same period, a large series reported a perinatal mortality rate of 1.8 per 1000 following ECV.^42^ These figures suggest near parity in neonatal outcomes between cephalic birth following ECV and breech birth.

The cost-effectiveness of breech care options in current UK maternity care requires investigation. Clinical and cost-effectiveness of ECV is sensitive to ECV success rates within a given setting, which vary from 14-49% in the UK.^40,43^ The presence or absence of support for vaginal breech birth, which also varies considerably, will also influence this. In Denmark, Jensen and Wüst^44^ found that, following publication of the last Term Breech Trial,^45^ the increased CS rate between 2001-2004 resulted in 3.5% higher baseline costs for breech babies, equivalent to 1.5 million dollars, including follow-up to two years. A recent cost-effectiveness analysis concluded that universal ultrasound to detect breech presentation would potentially be cost-effective in the NHS,^43^ but Wastlund et al’s model was based on elimination of all vaginal breech births. PPI work and available research^1–3,5,7,8,11^ indicate this is not what women want, so a model which accounts for planned breech births is needed.

None of the available trials or reviews includes studies of physiological breech birth (PBB) methods.^38^ A physiological approach to vaginal breech birth includes (but does not limit women to) upright, active birth positions. Louwen *et al*reported on 229 upright breech births compared to supine deliveries. Upright births were associated with significantly fewer manoeuvres (manual interventions, including forceps) (OR 0.45, 95% CI 0.31-0.68) and neonatal birth injuries (OR 0.08, 95% CI 0.01-0.58).^23^ Second stages of labour were 42% shorter, with a non-significant decrease in serious perineal lacerations. Rates of CS in labour also decreased following the implementation of upright breech birth. In Bogner *et al*’s smaller study of 41 matched pairs, upright breech births were associated with a reduction in severe perineal injury from 58.5% to 14.6%, and 70% of all births took place spontaneously, without the need for manoeuvres.^46^ Upright breech birth falls within the scope of current RCOG guidance^16^ but is less commonly used than traditional supine assisted delivery techniques. If more commonly available, these improvements may impact the clinical and/or cost-effectiveness of current care recommendations for breech presentation at term and women’s experiences of care.

Many studies are done about specific interventions in the care pathway for breech presentation in late pregnancy (>32 weeks), but few studies collect information about the entire care pathway. This includes trials of methods of turning the baby, drugs used to relax the uterus or provide pain relief when trying to turn the baby, gestational age at which to try turning the baby, methods of delivering the baby, organisation of services to deliver the baby, prediction of risk when turning or delivering the baby, and more. Very few of these studies will explore the interaction of these interventions with other aspects of the woman’s care, and even fewer of them will explore long-term outcomes for mothers and babies or economic implications.

The OptiBreech Care Pathway is a proposed innovation package based on programme theory developed from previous research, briefly summarised as follows:

- Providing reliable, experienced support for physiological breech births (OptiBreech Care, **the intervention**), in which women are encouraged to remain active and adopt the birthing position of their choice, will improve ***access to*** and ***outcomes*** of breech births
- because this is more acceptable to women than standard care,^4^ in which skill levels are unpredictably variable and low overall^47,48^ (**mechanism 1**);
- and because birthing in upright positions results in shorter labours and fewer interventions, compared to the supine birthing positions prescribed in standard care^38^ (**mechanism 2**);
- but these potential benefits may only be realised in contexts where specialist midwives and/or obstetricians are enabled to self-organise to support the wider maternity care team as required^49^ (**context**).

A full logic model for the OptiBreech Care Pathway, based on the Medical Research Council guidance for process evaluation of complex interventions^50^ is included below. A similar package of care is partially implemented in some sites within the UK^21,51^ and internationally.^52^ But we do not know if this care pathway is as clinically and cost-effective as standard care.

## Feasibility Testing of the OptiBreech model of care so far

We have conducted an evaluation of physiological breech birth training, one of the required elements of OptiBreech care, in six NHS hospitals in England and Northern Ireland.^53^ In addition to significant changes in confidence and knowledge, the training was associated with change in the use of upright birthing positions in practice (32% vs 81%, *p* = <.0001), so we know that this training leads to measurable changes in clinical practice.^53^ The study was not powered to detect differences in clinical outcomes, but the initial results were reassuring enough to continue with further research. Among births attended by staff who attended the training, there were no adverse outcomes, compared to 7% maternal and 7% neonatal adverse outcomes among births where no PBB trained professional was in attendance (0/21 versus 5/69, both maternal and neonatal). Rates of episiotomy were lower (5% versus 22%), and rates of intact perineum were higher (52% versus 39%). These are all outcomes that matter very much to women, and it is important that we evaluate them with adequately powered studies.

Table 1: Results of Physiological Breech Birth training

*Results of vaginal breech births only. Conducted in 6 hospitals across the UK. No attempt was made to quantify the experience/proficiency of attendant, only their completion of training package.*

| *Total = 90* | **PBB trainee**  **at the birth** (n=21) | | **No PBB trainee**  **at the birth** (n=69) | |
| --- | --- | --- | --- | --- |
|  |  |  |  | |
| **Birth Position** |  | |  | |
| upright | 17/21 | **81%** | 22/69 | **32%** |
| supine | 4/21 | **19%** | 47/69 | **68%** |
|  |  | |  | |
| **Maternal Severe Adverse Outcomes** | 0/21 |  | 5/69 | **7%** |
| PPH > 1500 mL | 0/21 |  | 3/69 | **4%** |
| 3^rd^/4^th^ degree tear | 0/21 |  | 2/69 | **2%** |
|  |  | |  | |
| **Perineum** |  | |  | |
| Intact | 11/21 | **52%** | 27/69 | **39%** |
| Episiotomy | 1/21 | **5%** | 15/69 | **22%** |
|  |  | |  | |
| **Neonatal Severe Adverse Outcomes** | 0/21 |  | 5/69 | **7%** |
| 5 min Apgar < 4 | 0/21 |  | 4/69 | **6%** |
| NICU > 4 days | 0/21 |  | 1/69 | **1%** |
|  |  | |  | |

*PBB trainee at the birth = Someone present at the birth who participated in Physiological Breech Birth training*

*PPH = postpartum haemorrhage; NICU = neonatal intensive care unit*

The OptiBreech 1 opened in January 2021, with 10 sites gradually joining the study throughout 2021 due to delays with R&D approvals affected by the COVID-19 pandemic (https://optibreech.uk/participating-sites/). This is a preliminary observational and qualitative feasibility study, aiming to determine whether there is a demand for vaginal breech birth, whether it is possible to implement the OptiBreech model of care, and how acceptable the OptiBreech model is to women and staff. As of 9 September 2021, a total of 27 women requesting a vaginal breech birth have been recruited to the study across 4 sites. A brief summary table is presented below, comparing our returned results so far, alongside the largest reported study of ECV outcomes in the UK.^40^

Table 2: Results of OptiBreech 1

*OptiBreech 1* – conducted in the hospitals participating in this randomisation study (to 7/9/21)

| **Outcome** | **OptiBreech 1**  *planned vaginal breech births following or without ECV attempt* | **Melo et al 2019 ECV study**  *gold standard UK ECV pathway* |
| --- | --- | --- |
| Total vaginal births | 48% (13/27) | 43.6% (1141/2614) |
| Spontaneous vaginal birth | 44% (12/27) | 33.1% (866/2614) |
| Instrumental birth  *(forceps or suction cup)* | 4% (1/27) | 10.5% (275/2614) |
| Pre-labour caesarean birth | 19% (5/27) | 36.6% (957/2614) |
| In-labour caesarean birth | 33% (9/27) | 18.5% (484/2614) |
| Baby turned head-down prior to labour following failed ECV | 4% (1/27) | 4% (57/1334) |
| Admission to NICU / SCBU* | 4% (1/27) | 3.6% (95/2614) |
| Stillbirth or neonatal death (within 28 days of birth) * | 0 | 0.19% (5/2614) |
| Someone present who had completed physiological breech birth training | 83% (10/12) | *Not reported* |
| Someone present who met all proficiency criteria | 75% (9/12) | *Not reported* |
| Less than 5 minutes elapsed between the birth of the pelvis and the birth of the head | 92% (11/12) | *Not reported* |
| Maternal birth position | Upright – 83% (10/12)  Supine (back) – 17% (2/12) | *Not reported* |

* The OptiBreech 1 study has not collected enough data to make a comparison on these outcomes, but we would expect results to be similar, based on the RCOG Guidance described below.

Although it is too early to conclude anything from these results, a few observations can be made. Firstly, where a genuine specialist midwife is in operation and attending most/all of the vaginal breech births, as is/was the case in two of the sites, there appears to be a significant demand for this service. These two Trusts, comprising a total of three hospitals, have been responsible for 21/27 of the women recruited in the OptiBreech 1 study. They are the only two sites to meet the inclusion criteria for OptiBreech Care. There has been 100% proficient attendance at these sites and no neonatal admissions.

In contrast, attempts to set up a multi-disciplinary team at other sites have been largely unsuccessful or are progressing slowly. This may be because the implementation approach used is not adequate/feasible or because the demands of dealing with the pandemic have hampered the ability to develop specialist skills within a service where they are not already operational. For example, several sites have indicated face-to-face training is needed, but this has been very challenging to arrange due to social distancing measures in place and persistent staff shortages. All adverse outcomes and lack of attendance of proficient OptiBreech team members have been in sites not currently included in this randomisation pilot. We will continue to observe what is happening in these sites to gain insight over a longer period of time, but no site will be able to randomise women in this study until the site eligibility criteria have been met.

Finally, our team has completed a review of outcomes associated with effectiveness studies of breech birth at term.^54^ This included significant involvement of our PPI group, to ensure that women’s voices influenced our choice of outcome data points. The influence they have had on our work is described in the publication.

## Patient and public involvement (PPI)

In preparing the funding application, feedback was sought from service users. This included the Fast Track Review offered by NIHR Research Design Service London of the Plain English Summary, a review of the entire application by the Birth Trauma Association and meetings with local Maternity Voices Partnership groups. Additionally, a PPI group was formed of women who had experienced a breech pregnancy in the UK within the last 5 years, with outcomes of birth after ECV, CS and vaginal breech birth represented. Invitations were circulated via social media. Over 30 women have been involved with PPI work in this project, along with the leaders of the groups we contacted. We have input from a variety of perspectives and have sought out involvement from women with black and minority ethnic backgrounds.

A summary of the proposal has also been presented at multiple breech training days, inviting discussion and written feedback from maternity care professionals. Summaries of feedback are available on-line via the feasibility study website (<https://optibreech.uk/category/ppi/>) created to share the development of the project with various stakeholders.

Women with a strong desire for vaginal birth and minimal intervention appear to be least satisfied with current care and have the greatest need for more evidence to underpin shared decision-making. Women described feeling ‘very pressured by doctors’ in their pregnancies, consistent with available research. They did not necessarily want research to tell them which option is ‘best.’ Some were wary that if PBB proved ‘safe,’ women would feel just as pressured to attempt a PBB as women currently feel to accept an ECV or CS. Women prioritised individualised care. They wanted to know if it was reasonably safe to make the choice which meets their unique preferences, values and circumstances, or if one option really was significantly better than another.

PPI work influenced the decision to include 2-year health usage and quality of life outcomes at the feasibility phase. Women have been disappointed by a focus on short-term outcomes and a disregard for long-term outcomes, including those for future pregnancies.^55^ Research also indicates these are most important to women.^18,54^ These longer-term outcomes require more planning from the start of a study in order to successfully collect.

Women who participated in our PPI had a strong wish to contribute to a better research base for breech birth, but they also wanted reassurance no one would suffer distress due to randomisation. This influenced the decision to choose a pragmatic trial design.^56^ The feasibility Trial Within a Cohort (TWiC) design^57^ compares a model of care delivery designed to support vaginal breech birth as safely as possible (OptiBreech), with standard care, designed to minimise vaginal breech births through ECV and CS, but it does not require women to submit to one treatment or another. Women who do not wish to be randomised are able to contribute their data through participation in the cohort study, which will enable the research team to collect a larger amount of prospective, intention-to-treat data for more rare safety outcomes. Women who are randomised are also able to change their minds. This most closely replicates what happens in ‘real life’ in both of these models.

Involvement has also been sought from key hospital clinical and research staff throughout the UK. Their views also influenced the pragmatic trial design. They identified that demand for ECV, CS and breech birth is influenced by local cultural and media influences, so a restrictive, explanatory trial design could limit regional participation in a multi-centre trial and subsequent generalisability. In May and October 2019, we gathered feedback from 130 professionals from participating Trusts and piloted an acceptability questionnaire (<https://optibreech.uk/2019/06/06/what-do-staff-think/>).

Throughout the OptiBreech 1 study (IRAS 268668), we have engaged with our PPI group and professional stakeholders through virtual events.^55^ This has focused on development of the study design, for example processes for when a breech presentation is diagnosed for the first time in labour, and study documents, such as consent forms and participant information sheets/videos. We also held an engagement event to enable our PPI group to provide feedback on the NICE Antenatal Care guideline development.

# Aim, objectives and outcomes

This research asks: 1) Is it feasible to conduct a randomised trial within a cohort (TWiC) comparing OptiBreech Care for planned vaginal breech birth with external cephalic version, for women with a breech pregnancy at term?

The aim is to determine whether a TWiC is feasible and offers value for a future policy change.

## Objectives

**Cohort study:**

1. Pilot the OptiBreech cohort database using data from women participating in the OptiBreech 1 study, who have already given prospective consent for their data to be collected;
2. Measure the completeness of outcome data and time required to gather it;
3. Identify preliminary safety outcomes among a larger cohort of women planning a vaginal breech birth with OptiBreech Care, some of whom may not be eligible for randomisation.

**Trial delivery and design:**

1. Identify recruitment, adherence and retention rates to inform estimations for a full RCT;
2. Determine outcomes to be prioritised and sample size for a full RCT;
3. Determine with a Trial Steering Committee whether an RCT is feasible and offers value for a future policy change.

**Implementation evaluation:**

1. Assess the fidelity of the OptiBreech Care Pathway delivery within the cohort, based on the TiDIER checklist^58^;

**Economic evaluation:**

1. Identify the relevant resources and health services used and test appropriate methods for their measurement; and
2. Determine which costs and benefits to the NHS are feasible to measure in a full trial.

## Primary Outcomes

1. Recruitment rate recorded as the number of eligible participants who consent to participate in the study by 6 months (randomised) and overall (non-randomised);
2. Acceptance rate recorded as the number of participants randomised to OptiBreech Care who plan a vaginal breech birth, and the number of participants randomised to the control who attempt an ECV, measured at the time of birth;
3. Attrition rate recorded as the number of participants who consent to participate who remain in the study until the end of follow-up at 4 months after birth;
4. Long-term attrition rate recorded as the number of OptiBreech 1 participants who complete 1-year and 2-year follow-up surveys when invited;
5. Fidelity to intervention recorded as number of planned VBBs attended by a proficient team member, measured at the time of birth;
6. Costs to deliver the service recorded as total number of days and nights spent on call to support planned VBBs in the trial by 6 months.

## Secondary Outcomes

The following potential primary and secondary outcomes for a substantive trial will be feasibility-tested, and incidence rates will be used to inform power calculations for the substantive trial.

1. Admission to higher-level neonatal care, measured at 28 days following birth, as a binary (yes/no) and continuous (number of days/nights) outcome, from patients’ medical records;
2. Mode of birth measured using patient’s medical records on day of birth, as a categorial measurement to include the following categories: vaginal breech birth, forceps breech, pre-labour CS, emergency CS, cephalic vaginal birth, cephalic forceps, cephalic ventouse;
3. Composite neonatal perinatal death or serious adverse morbidity, measured at 28 days following birth, from patients’ medical notes; serious neonatal morbidity to include the following: 5 minute APGAR score <7, peripheral nerve injury present at discharge from hospital, skull fracture, spinal cord injury, admission to NICU>4 days, intubation/ventilation >24 hours, convulsions >24 hours, parenteral or tube feeding >24 hours;
4. Composite maternal death or serious morbidity, measured at 28 days following birth, from patients’ medical notes; serious maternal morbidity to include the following: postpartum haemorrhage >1000 mL, obstetric anal sphincter injury, cervical laceration involving lower uterine segment, vertical uterine incision or serious extension to transverse uterine incision, bladder, ureter or bowel injury requiring repair, dilation and curettage for bleeding or retained placental tissue, manual removal of placenta, uterine rupture, hysterectomy, vulval or perineal haematoma requiring evacuation, wound dehiscence / breakdown, wound infection requiring prolonged hospital stay / readmission / antibiotics, sepsis, disseminated intravascular coagulation;
5. Use of services following referral for breech care, to include antenatal and postnatal appointments, total time spent admitted to hospital, number of ECVs, number of ultrasound scans, and professionals present at birth, measured at 28 days following birth from patients’ medical notes;
6. Satisfaction with care, measured using previously validated survey questions with a 5-point Likert scale, at 1 month post birth
7. Experience of childbirth, measured using the ‘Childbirth Experience Questionnaire’^59,60^ at 1 month post birth
8. Health-related quality of life, using the PROMIS-10 survey^61^ at 1 month, 3-4 months, 1 year and 2 years following birth
9. Infant’s development, using the appropriate Ages and Stages Questionnaires at 3-4 months, 1 year and 2 years following birth^62^

# Study design and Flowchart

This study uses a Trial Within Cohort (TWiC) design. That is, some of the women contributing their data will be randomised and others will not be.

## The OptiBreech Multiple trial cohort (OptiBreech mTC)

This study will establish and use a cohort database for a long-term prospective observational study of OptiBreech care. That is: care for breech presentation at term based on the principles of physiological breech birth and delivered by specialists with a specified minimum level of training and proficiency. Once refined, the intention is that multiple centres delivering this type of care will be able to contribute data in order to establish more robust and generalisable measurements for safety outcomes. A majority of studies of care for breech presentation at term are small, single-centre observational studies, which often do not involved numbers large enough to evaluate the questions that are most important to service users, such as the current perinatal mortality, serious morbidity and long-term impairment. Collecting observational cohort data, among women who decline or are ineligible for randomisation, or within sites that are not yet participating in the randomisation element, will enable the results of the randomised study to be contextualised.

The OptiBreech database will be piloted by collecting the full data set from the medical notes of women who participated in OptiBreech 1. These women have already given consent for their data to be used and identifiable information to be retained for future research. We will pilot the case report form (CRF) and the on-line database by retrospectively collecting their data and entering it into the database. They will be an internal pilot, included in the non-randomised cohort group. Refinements will be made to the CRF and/or database to improve the quality of the dataset we are able to obtain for the OptiBreech mTC.

## The OptiBreech-ECV Trial

This study will evaluate the feasibility of comparing OptiBreech care with the standard care pathway, offering and encouraging ECV, by randomising women within the cohort who consent in a pilot trial. The results will be compared with each other, and to the outcomes for women within the cohort who were otherwise eligible for but did not consent to randomisation. It is a pragmatic trial designed to compare the care pathways as they would be delivered were the OptiBreech model to be implemented as policy. Women and birthing people who participate will retain their usual rights to decline the care offered.

## Flowchart

Figure 1: Study Design Flowchart

## Research Timeline

Figure 2: Research Timeline

# Participant Selection

## Sites

All NHS sites who are providing OptiBreech Care are eligible to contribute data to the cohort study.

Site requirements for participation in the pilot randomisation include:

1. Recruited a minimum of 5 women to OptiBreech 1 or the non-randomised cohort of OptiBreech mTC
2. Achieved at least 90% fidelity to the intervention (attendance of someone with training and/or proficiency, <5 minutes from pelvis to head and use of maternal movement and effort prior to hands-on assistance) for a minimum of 5 vaginal breech births
3. Able to present a plan for recruitment and randomisation for a minimum of 2 women per month who provide fully informed consent to taking part in the study, immediately following diagnosis of breech presentation by USS, prior to counselling for ECV/mode of birth
4. Able to ensure a member of the OptiBreech team is available to provide counselling for women randomised to ‘offer OptiBreech Care’
5. Able to include the Physiological Breech Birth Algorithm in annual mandatory training activities for all staff. This should cover: The recommended 7-5-3 minute time limit guidance; actions in response to delay in second stage, including delay on the perineum; physiological neonatal transition, including initiation of resuscitation where required with umbilical cord intact; effective communication; and maintaining a helicopter view
6. Identification of a Breech Specialist Midwife and/or Obstetrician with dedicated time to co-ordinate OptiBreech care and training, ability to deliver the physiological breech birth training package and commitment to participate in on-going professional development seminars provided by the research team throughout this trial.

Sites will be able to demonstrate adherence to these criteria during their participation in the OptiBreech 1 study.

We will initially include 2 Trusts, including 3 hospital sites, in the randomisation pilot. Approximately 4% of women have a breech pregnancy at term, and we can expect at least half of these to be eligible for randomisation. Feasibility testing as part of OptiBreech 1 indicated that we can anticipate half of these again, or 1% of all women, will consent to randomisation in this study. Sites will be expected to recruit a minimum of 10 participants willing to be randomised over a 6 month period.

## Participants

### OptiBreech mTC – Cohort inclusion criteria

Eligibility to participate in the cohort study will include:

- Live, singleton pregnancy with a breech-presenting fetus confirmed by ultrasound scan;
- Over 16 years of age;
- Referred for specialist care for breech presentation antenatally from 32 weeks;
- Breech presentation from 37 weeks discovered in labour;
- Requesting or preferring a vaginal birth; and
- Giving informed consent to participate to contribute data to the cohort study. *(Note: For women in active labour, consent should be sought AFTER the birth; see below.)*

### Cohort Exclusion criteria

The following will be excluded from the cohort study:

- Absolute reason for caesarean section already exists (e.g. placenta praevia major);
- Requesting a caesarean section prior to recruitment;
- Multiple pregnancy;
- Life-threatening congenital anomaly; or
- Not consenting to contribute data to the cohort study

### OptiBreech-ECV Trial – Additional inclusion criteria for randomisation

In addition to the cohort inclusion criteria, eligibility for randomisation will include:

- Consent to randomisation.

### Trial exclusion criteria

Exclusion criteria include any contraindication listed in the RCOG guidelines for external cephalic version and/or management of breech presentation at term, including:

- Has already had an ECV attempt prior to recruitment
- Rhesus isoimmunisation
- Current or recent (less than 1 week) vaginal bleeding
- Evidence of antenatal fetal compromise, including abnormal electronic fetal monitoring
- Rupture of the membranes
- Hyperextended neck on ultrasound
- Estimated fetal weight less than 2000 g or less than 10^th^ centile at recruitment (if a growth scan has been performed)
- Estimated fetal weight greater than 3800g or over 95^th^ centile at recruitment (if a growth scan has been performed)
- Standing / footling presentation at the time of recruitment, defined as hips extended and breech above the inlet to the pelvis or not longitudinal;
- Any indication at the time of recruitment for induction to be recommended prior to 41 weeks of pregnancy, e.g. gestational diabetes, obstetric cholestasis, advanced maternal age;
- Breech diagnosed for the first time in labour; and
- 2 or more previous caesarean sections.

# Study procedures

## Participant recruitment

OptiBreech cohort sites should establish their own pathways for screening, approaching and consenting potentially eligible women and birthing people. An ideal pathway would look like this (Figure 1):

1. Breech presentation is suspected on palpation. The woman is referred for an ultrasound scan to confirm. When a clinician makes a referral, the woman should be directed to the information, including the approved Participant Information Sheet, available on the feasibility study’s website (https://optibreech.uk).
2. Women booked for a presentation scan are initially screened by a member of the clinical team providing direct clinical care, and those eligible for recruitment are flagged.
3. Breech presentation is confirmed on ultrasound. Immediately after this scan, the woman is asked if she would like to participate in the study. She is offered an opportunity to ask questions and consent is obtained, by the staff member if authorised to collect consent on the delegation log, or a member of the research team.
4. If the woman is also eligible to be randomised to standard care or OptiBreech care, based on the consents she has given and her scan results, she is randomised. The woman should receive a copy of all relevant Participant Information Sheets and a copy of her fully executed consent form.
5. She begins the care pathway she is randomised to. A copy of the GP letter, relevant Participant Information Sheet(s) and Consent form is sent to the GP.

This is ideal because OptiBreech care is a care pathway intervention, and the care pathway begins when the woman is first counselled.

Eligibility for randomisation among the cohort participants is automatically captured in the eCRF during the recruitment process. We are opting not to use an additional screening log, although individual sites may wish to create their own, as this information is already automatically captured on Edge. Having an additional log has not been shown to increase recruitment but does add significantly to the research team’s workload.^63^ Instead, we will monitor site activity through absolute recruitment and adherence rates. We will use a delegation log, and all local research team members involved in recruitment should be listed on this log once the local PI has ensured they have received training. As this is an intervention study, all those taking consent for participation in the study should have current GCP training in place, or have completed the OptiBreech training that includes GCP-lite training relevant to this study, and be listed on the delegation log.

Consent will be completed directly through the study’s on-line database, provided by MedSciNet. The consent form is available in an electronic format on the study database to which participants have direct access, and they will sign consent online. Access will be provided via the e-mail address given by potential participants for this purpose. The system will confirm who accessed the database and the date. The consent form will be countersigned by separate access by the person taking consent, so that the data and time are recorded. This may be completed face-to-face or asynchronously through the online access provided. A fully executed version is saved within the database. A copy is exported to be stored in the participant's medical record, and the participant can download a copy for their own records using their direct access.

Consent will ideally be a research midwife, but because consent is taken prior to the start of their breech care pathway, it can be taken by any healthcare professional who has completed GCP training or OptiBreech GCP-lite training and signed the delegation log. When women are enrolled, they will be given login information so that they can enter their details directly onto the database. These login details will later be used to complete the follow-up surveys if women have consented to receive these. Consent will be confirmed on-line by the health care professional who has taken consent. Paper copies of the PIS and Consent forms will be provided in case the on-line version fails or is inaccessible.

A copy of the consent form will be sent to the woman and local research team, to be filed in the woman’s notes. Where paper-based consent forms have been used, a scanned copy should be sent by secure electronic transfer or encrypted NHS e-mail to the core research team.

PPI work indicated that enabling women to access participation in this study if they wish was a priority. All PPI group participants had experience of either their own difficulty seeking support for a vaginal breech birth, or experience of supporting women who were having difficulty accessing care for a vaginal breech birth. Additionally, approximately 1/3 of the participants in OptiBreech 1 were self-referrals, who transferred care from another Trust to receive support for a vaginal breech birth within a hospital participating in OptiBreech 1. Therefore, if a Trust feels it is ready to accept self-referrals, contact information for their Breech Clinic will also be made available via the study’s website, for women who may wish to self-refer. Women may self-refer either internally or from a different Trust if they would like to participate in the research. In this case, women would be screened by OptiBreech staff for suitability.

The minimum recruitment target for each site in this 6-month pilot trial is 10 women who consent to randomisation. Each additional recruitment to the cohort study (non-randomisation) will count as an accrual, but the minimum recruitment target is based on women who consent to randomisation.

### Breech presentation diagnosed in Labour

Within sites where routine 3^rd^ trimester scans are not offered, breech presentation is diagnosed for the first time in labour among 20-30% of the population.^64,65^ Almost all of these would be eligible for the OptiBreech Cohort, although not randomisation. Therefore, it is appropriate to include their outcomes within the cohort, if consent is given.

In these cases, the potential participant will be informed about the study approximately 24 hours after the birth during a debrief of the birth itself. They will then be asked for consent to include their data. Where care recipients do not provide consent, their data will not be used. Local PIs should establish a system to identify unplanned VBBs so that they can be included in the cohort study.

This procedure is consistent with the current RCOG guidance on “Obtaining Valid Consent to Participate in Perinatal Research Where Consent is Time Critical.”^66^ It was specifically discussed at a PPI group meeting on 15 July 2020 and modified based on the group’s feedback to ensure the request for consent was also accompanied by a full debrief of the woman’s birth experience. Women in the group who had experienced an undiagnosed breech birth felt this would have been particularly helpful to them, resulting in a consent process that was both beneficial for the women themselves as well as for the research. We have piloted this procedure in OptiBreech 1.

Completion of the debrief is monitored through the CRF.

Where an adverse outcome has occurred during an unplanned vaginal breech birth, it is important to include these outcomes in the study, subject to consent, whether an OptiBreech team member was present or not. However, this requires very sensitive counselling, and the woman may wish to decline follow-up surveys. This should be offered by the Breech Lead Obstetrician or Breech Lead Midwife and further referrals for counselling and support should be made as appropriate.

## Screening Procedures

The clinical team with a duty of care for the woman should review the potential participant’s medical record prior to the scheduled ultrasound scan appointment to screen for eligibility, and if eligible, ensure they have information about the research prior to attending. They should also ensure research staff are alerted and available to take informed consent. If the woman is eligible for the cohort and consents to randomisation, the eCRF will guide the research team member through the eligibility criteria.

However, one of the challenges to studying breech care is the variety of times at which women are diagnosed and/or referred for care. In some Trusts, women are referred for care from as early as 32 weeks so that they can be counselled about using moxibustion and/or postural interventions to encourage baby to turn head-down. In some pregnancies, the breech presentation is not diagnosed until an ultrasound scan is performed for other reasons at a later gestation. Some breech presentations remain undiagnosed until the woman is in labour. And some women will transfer care to access support for a vaginal breech birth after multiple failed ECV attempts elsewhere. In some instances, the circumstances will mean the women are ineligible for randomisation but can be included in the cohort. In the first two instances, women may still be eligible for randomisation and should be offered it as early as possible in their care. Having had an ECV in this pregnancy, prior to recruitment, is an exclusion criteria for randomisation but not the cohort should women be identified as eligible later than the idea recruitment point.

Because of this variability, all clinical staff who may encounter women at these various stages should be aware of the research and how to refer to the research team. This will include all clinical staff providing ultrasound scans for breech presentation, such as midwives, obstetricians and sonographers. And any staff referring women for scans should provide information about the research in advance.

## Randomisation Procedures

A total of 104 participants eligible for randomisation, who have consented to randomisation, will be automatically randomised to either OptiBreech Care or the control (standard care). The randomisation schedule will be computer-generated, using MedSciNet software. Allocation will be automatic during the enrolment process on the database and revealed to the person who is taking consent and enrolling the participant onto the study. All local research team members who are authorised to take consent will have login information to complete this process. Therefore, randomisation can only occur when one of them is available, at the earliest opportunity after the breech presentation is diagnosed.

Minimalisation factors will include site, parity (0 vs 1 or more previous births), type of breech presentation (extended/frank vs any other), and gestation at enrolment (<36 weeks, 36-38+6, 39+ weeks). Allocation between arms will be equal. The enrolment log will be completed automatically through the database.

Due to the two-stage consent process developed in collaboration with the PPI group, women randomised to the control will be cared for within the standard local care pathway, without any further steps in the consent process.

Women randomised to OptiBreech Care will be informed they have been randomised to OptiBreech Care, given the OptiBreech Care Information Sheet (paper or on-line version), and scheduled for counselling by a member of the OptiBreech team as per the Description of Intervention below. Consent for mode of birth is a standard part of all antenatal care, and national guidelines recommend that women should be offered the option of a vaginal breech birth, so further specific consent to receive specialist OptiBreech care will not be taken. Instead, women will be informed that they can request to see another member of the team at any point.

## Masking and other measures taken to avoid bias

### Masking

It is not possible to mask participants randomised to OptiBreech Care due to our duty to describe the potential risks and benefits of a non-standard care pathway to those offered it. However, their allocation will not be indicated on their hand-held CRF or medical notes. Staff attending births and conducting subjective judgements, such as the baby’s Apgar score at birth, may know if a woman has had an attempt at ECV and is enrolled on the OptiBreech study. However, they may not know which arm the woman has been randomised to, due to the pragmatic nature of the trial. Some women will be in the cohort but not randomised, others will be randomised to one arm but choose the recommended treatment in another arm (ECV or planned VBB). Therefore, analysis by intention to treat will minimise some of the bias. All data analysis will be undertaken blind to allocation.

## Schedule of treatment for each visit

Below is a treatment schedule that represents our best prediction of what is likely to happen, based on our initial feasibility testing in OptiBreech 1. The nature of individualised care in a complex intervention, and the nature of breech presentation itself, means that the actual number of visits may vary. The schedule below corresponds to the activities listed on the SoECAT form.

| Visit | Standard Care | OptiBreech Care |
| --- | --- | --- |
| Screen / Diagnosis Day 0 | Bedside ultrasound scan  Participant consent | Bedside ultrasound scan  Participant consent |
| Visit 1, Counselling | Detailed ultrasound scan with sonographer  Counselling  Consent for ECV* | Detailed ultrasound scan with sonographer  Counselling  Consent for mode of birth* |
| Visit 2, External cephalic version | External cephalic version  Standard antenatal observations  Cardiotocograph monitoring  Tocolytic prior to procedure |  |
| Visit 3, Follow-up | Bedside ultrasound scan  Standard antenatal observations  Consent for mode of birth | Standard antenatal check-up with OptiBreech team, review of birth plan |
| Visit 4, Care in labour | Standard labour care as planned | Attendance of experienced practitioner during 2^nd^ stage of labour (minimum), in addition to standard labour care as planned |
| Visit 5, Follow-up |  | Debrief with birth attendant |

The above list of visits represents the ‘typical’ or ‘ideal’ breech care pathway in each arm of the study. However, these may vary according to maternal choice, for example where women in the ECV arm of the study choose not to have an ECV and opt for a VBB or CS instead, or where women request an ECV prior to a VBB attempt or CS in the OptiBreech arm. Women in each arm will continue with usual antenatal care as indicated by local guidelines. The schedule above represents additional care related to breech presentation.

## Follow-up Procedures

Participants in this study are followed up by surveys, conducted at 1 month, 3-4 months, 1 year and 2 years after the birth of their baby. They will be contacted by the OptiBreech KCL research team using the e-mail address they provide. They will answer the surveys online, and the information will be directly entered into the OptiBreech mTC Database and associated with their unique ID. To feasibility test the procedures within the timeline, longer-term follow-up surveys will be completed with women participating in the OptiBreech 1 study, who have already given their consent to follow up. They will be contacted by the KCL research team directly, and this will not require further involvement from local research teams.

## Radiology assessments

All radiology assessments included in the study are listed above. They are a standard, necessary part of diagnosis and assessment of breech presentation in pregnancy, regardless of to which care pathway the person is randomised. No additional radiology assessments are indicated solely for the purposes of the study.

## End of Study Definition

Recruitment for this study will be complete when 104 women have been recruited to the OptiBreech-ECV pilot trial component of the feasibility study. There is no minimal number required to be recruited to the observational component. The REC will be informed that the study has completed when the TSC has reviewed the data and issued their opinion on the feasibility of a full RCT.

# Description of intervention

*OptiBreech Care* is a care pathway intervention^67^ that starts at the point of diagnosis of breech presentation and referral to specialist care and continues until birth. The way in which OptiBreech care differs from standard NHS care (‘offer ECV’) is outlined below, using a TiDIER checklist.^58^

## Tidier Checklist

Table 3: TiDIER Checklist: Comparison of standard care (‘offer ECV’) with the Intervention (‘offer OptiBreech vaginal breech birth care’)

| **Item Number** | **Item** | **Description** | | **Where located / Reference(s)** | **How fidelity is measured** |
| --- | --- | --- | --- | --- | --- |
|  |  | Standard NHS Care | OptiBreech Care Pathway |  |  |
| 1 | Brief Name  Name or phrase that describes the intervention | Offer ECV | Offer OptiBreech care | *EPOC: Care pathways aim to link evidence to practice for specific health conditions and local arrangements for delivering care.* | Has the woman been offered ECV?  Has the woman been counselled by an OptiBreech team member? |
| 2 | Why  Describe any rationale, theory or goal of the elements essential to the intervention | Current standard of care. Follows current RCOG and NICE guidance.  The goal of external cephalic version is to turn the baby to a head-down position in the womb. This is expected to make a vaginal birth more likely and safer because the baby is head-down. | OptiBreech care is continuity of care by a breech-proficient team, led by a Breech Specialist Midwife and a Breech Lead Obstetrician. All care is co-ordinated by the Breech Specialist Midwife.  The Specialist Midwife meets all OptiBreech proficiency criteria and ensures that they or a similarly proficient member of the team attends all planned vaginal breech births.  Current RCOG guidelines state attendance of ‘skilled and experienced’ professional may make vaginal breech birth nearly as safe as cephalic birth.  Women prioritised knowing how safety of VBB compares with cephalic birth following ECV, which was their alternative. | Rationale outlined in protocol introduction and description of intervention | Attendance at all planned OptiBreech births by a member of staff who meets proficiency requirements.  Proficient OptiBreech attendants are registered on the delegation log. Professionals maintain their own portfolio and self-report training and proficiency criteria. |
| 3 | What  **Materials:** Describe any physical or informational materials used in the intervention, including those provided to participants or used in intervention delivery or in training of intervention providers. | Care pathway follows the local guideline for the ECV service.  RCOG leaflet, Information for Women  Staff receive annual vaginal breech birth training, approximately 20-45 minutes per year, as part of an obstetric emergencies update. Most updates focus on supine methods of delivery only. | RCOG Information leaflet and information about the results of OptiBreech 1 to date.  Materials for professionals – Physiological Breech Birth training, a fully-evaluated training programme provided by Breech Birth Network, either in person or on-line.  Funding is provided for Breech Specialist Midwife time. They are expected to lead breech training in their institution, along with input from the Breech Obstetrician, and participate in on-going practice support workshops.  FaceBook peer support group for members of OptiBreech PPI group and study participants. | Educational content outlined in: Mattiolo & Walker, 2020, Physiological breech birth training: a multimethod pre-post intervention study, *Birth* (under review post-revisions)  On-line course is consistent and replicable. | Attendance at all planned OptiBreech births by a member of staff who has completed enhanced training.  Recorded on Pro Forma and Case Report Form |
| 4 | What  **Procedures:** Describe each of the procedures, activities, and/or processes used in the intervention, including any enabling or support activities. | Offer ECV  Some services may offer other advice re: turning the baby, such as moxibustion or postural exercises  If unsuccessful, offer caesarean section  If declined, care for vaginal breech birth is provided by the staff on duty when in labour, according to local guideline.  RCOG guideline recommends intervention is birth is not complete within 5 minutes of birth of pelvis or 3 minutes from the birth of the umbilicus.  Women may be referred for OptiBreech care if requested. | Offer management of vaginal breech births, according to Principles of Physiological Breech Birth, including caseloading by Breech Specialist Midwife and/or team.  Follow the OptiBreech Practice Guideline, in addition to the RCOG guideline.  Use of Physiological Breech Birth Algorithm to support decision-making in late second stage. The Algorithm recommends 7-5-3 minute time limits from +3 station, birth of pelvis and birth of umbilicus. Intervention is recommended sooner in order to remain within these limits.  If declined, offer ECV. Other advice, such as moxibustion or postural exercises is NOT offered unless vaginal breech birth with OptiBreech care is declined, or the woman requests information.  If declined, support caesarean section. | (Principles) Walker S, Scamell M, Parker P. Principles of physiological breech birth practice: A Delphi study. Midwifery. 2016;43(0):1–6.  (Algorithm) Reitter A, Halliday A, Walker S. Practical insight into upright breech birth from birth videos: A structured analysis. Birth. 2020;47(2):211-219. | Use of maternal movement and effort prior to hands-on intervention (principle), under ‘Fidelity’  Less than 5 minutes from birth of pelvis to head (Algorithm), under ‘Fidelity’  Documentation on Pro Forma. |
| 5 | Who provided  For each category of intervention provider (e.g. psychologist, nursing assistant), describe their expertise, background and any specific training given. | ECV is provided by an obstetrician or a midwife who has completed a programme of training and has been certified as competent to perform the procedure.  Counselling about mode of birth is provided by a member of the obstetric team.  Vaginal breech birth is expected to be supervised by the senior obstetrician on duty, unless delegated or otherwise agreed. | Women are counselled antenatally by the Breech Specialist Midwife or a proficient member of the OptiBreech team delegated by them.  Low-risk women, otherwise under midwife-led care, are not required to see an obstetrician for further counselling, unless further risks are identified. Their named consultant should be informed of their planned mode of birth.  The Breech Specialist Midwife or a proficient member of the OptiBreech team delegated by them is considered the lead for all intrapartum care, unless escalated and handed over due to complications. Their role is supervisory rather than hands-on.  All hands-on intrapartum care should be provided by someone who has received training in physiological breech birth, either through the training package or as part of annual mandatory training that includes this.  *Professionals’ proficiency is assessed using proficiency criteria in protocol | Proficiency criteria outlined in protocol  Walker S, Scamell M, Parker P. Standards for maternity care professionals attending planned upright breech births: A Delphi study. Midwifery. 2016;34:7–14. | Counselling is recorded on CRF.  Presence of a trained and/or proficient team member is recorded on CRF. |
| 6 | How  Describe the modes of delivery (e.g. face-to-face or by some other mechanism, such as internet or telephone) of the intervention and whether it was provided individually or in a group | Counselling regarding ECV is provided as per the Trust’s current guideline. This may be in a specialist clinic, in a standard antenatal clinic or ad hoc by the obstetrician on-call when breech presentation is diagnosed.  Proficiency in vaginal breech birth is self-assessed.  The local guideline for management of breech presentation should be followed. | Counselling re OptiBreech VBB care is provided by a proficient member of the OptiBreech team.  Proficiency is assessed and monitored by designated Breech Leads.  The OptiBreech Practice Guideline should be followed, referring to the local and national guidelines for anything not covered. | Outlined in Standards paper and  Walker S, Scamell M, Parker P. Expertise in physiological breech birth: A mixed-methods study. Birth. 2018:45(2):202-2009. | Counselling is recorded on CRF.  Experience and training reported on CRF. |
| 7 | Where  Describe the type(s) of location(s) where the intervention occurred, including any necessary infrastructure or relevant features. | Hospitals of various sizes throughout the UK  *(demographics to be described in detail in report)*  Guidelines recommend place of birth is obstetric unit, with lead professional the consultant obstetrician on duty. | Hospitals of various sizes throughout the UK  *(demographics to be described in detail in report)*  Guidelines recommend place of birth is obstetric unit, with lead professional a currently proficient member of OptiBreech team. | (Principles) Walker S, Scamell M, Parker P. Principles of physiological breech birth practice: A Delphi study. Midwifery. 2016;43(0):1–6. | Place of birth recorded on CRF |
| 8 | When and How Much  Describe the number of times the intervention was delivered and over what time period including the number of sessions, their schedule, intensity or dose. | Follows RCOG guidelines on external cephalic version and management of breech presentation at term.  Pathway begins when referred for specialist care for breech presentation. This can be as early as 32-35 weeks if units offer moxibustion prior to external cephalic version.  Participating units should not alter their current practice with regard to when women are referred for specialist care due to breech presentation, and there is no expectation to refer earlier than 36 weeks unless that is already current practice in the unit. | Pathway begins at 36 weeks. If women are randomised prior to 36 weeks because of earlier referral, they receive initial counselling about the research and an appointment to return at 36 weeks for review and care planning if still breech.  Minimum of one session of counselling and birth planning with a member of the OptiBreech team.  All intrapartum care should be provided by someone who has completed the training package.  Specialist Midwife or delegate attends as lead for the birth. |  | All episodes of breech care will be recorded on the CRF.  Number of days/nights someone has spent on call recorded on the CRF. |
| 9 | Tailoring  If the intervention was planned to be personalised, titrated or adapted, then describe what, why, when, and how. | Tailoring to women’s preferences is recommended in the RCOG guideline, but because mandatory training rarely includes upright birthing positions, these are less commonly used. | Upright birthing positions are a central component of physiological breech birth training. 80% of births are managed in upright positions. The aim is to facilitate women’s choice of birthing position rather than to dictate it. | (Evaluation) Mattiolo, S., Spillane, E., & Walker, S. (2021). Physiological breech birth training: An evaluation of clinical practice changes after a one‐day training program. *Birth*, birt.12562. https://doi.org/10.1111/birt.12562 | Variations such as place of birth outside the obstetric unit, maternal positioning, etc. will be described. |
| 10 | Modifications  If the intervention was modified during the course of the study, describe the changes (what, why, when and how) | Any modifications will be described in the report | Any modifications will be described in the report |  |  |
| 11 | How Well  Planned: If intervention adherence or fidelity was assessed, describe how and by whom, and if any strategies were used to maintain or improve fidelity, describe them. | Women are able to decline offer of ECV and may not adhere to recommended treatment. | Women are able to decline offer of OptiBreech VBB care and may not adhere to recommended treatment.  Adherence to OptiBreech guideline is promoted by use of an Algorithm and pro forma. | Walker S, Scamell M, Parker P. Expertise in physiological breech birth: A mixed-methods study. Birth. 2018:45(2):202-2009.  (Algorithm) Reitter A, Halliday A, Walker S. Practical insight into upright breech birth from birth videos: A structured analysis. Birth. 2020;47(2):211-219. | Adherence measured by number of women who accept/decline intervention to which they are randomised.  Proficiency assessed by breech leads and monitored through delegation log.  Adherence to Algorithm measured in pro forma and reported in CRF. |
| 12 | How Well  Actual: if intervention adherence or fidelity was assessed, describe the extent to which the intervention was delivered as planned. | (To be described in report) | (To be described in report) |  |  |

## Logic model

Figure 3: Logic Model for OptiBreech Care

## Proficiency criteria

A professional is considered currently proficient to facilitate OptiBreech care if they have:

1. Participated in 6 hours of evaluated physiological breech birth training;^53^
2. Attended at least 10 vaginal breech births, including resolution of complications using manual manoeuvres;
3. Attended or taught in simulation at least 3 vaginal breech births within the past year; *
4. Delivered physiological breech birth training at least once within the past year, including reflective reviews of births attended;
5. Completed an OptiBreech Proficiency self-assessment and indicated that they feel competent to implement the OptiBreech Practice Guideline at vaginal breech births where they are the designated clinical lead, and this has been confirmed by the OptiBreech Leads.

* Where professionals have attended at least 10 vaginal breech births in their career, but not 3 within the past year, it is possible to meet these criteria by teaching physiological breech birth simulations. This is because teaching skills involves more complex recall, including anticipation of others’ thought processes; and teaching has been identified in research as an important aspect of developing and maintaining proficiency.^49,68^

Additionally, while the nature of the research requires strict selection criteria for participation, these limits do not apply to the acquisition of breech birth experience. Therefore, teams may wish to consider additional ways for the clinical team to acquire and maintain proficiency, alongside the study cases and clinical teaching. These may include:

- Attendance at term breech births not included in the study;
- Attendance at multiples births involving at least one breech presentation;
- Attendance at preterm breech births; and
- Attendance at known stillbirths. In the case of intrauterine death, the presence of a skilled breech practitioner may minimise the trauma of a vaginal birth, which will more often need assistance due to lack of tone.^68^

In order to proceed with a vaginal breech delivery as part of the OptiBreech trial, it is mandatory for one fully proficient OptiBreech Team member to be present throughout the active second stage (i.e. from starting pushing, to completion of delivery), to offer support and maintain situational awareness. Hands-on clinical care should be provided by someone who has received physiological breech birth training as described above, either through completion of the OptiBreech training package or as part of annual mandatory training activities. If this level of training and experience is not available, the situation must be escalated to the on-call consultant to decide whether to discuss with the woman, whether a standard vaginal breech delivery can be considered or alternatively, whether delivery by caesarean section is required.

Ability to have a proficient team member attend 90% of planned vaginal breech births is a criterion for entering the randomisation component of this study. This will be confirmed either through participation in OptiBreech 1 or through enrolment of women requesting a VBB in the observational cohort study.

## Counselling

For women randomised to OptiBreech care, counselling regarding mode of birth will be undertaken or supervised by an OptiBreech team member who meets the proficiency criteria. This could be an obstetrician or midwife. Those team members providing counselling should be listed on the Delegation Log. This team member will put a plan in place, in collaboration with the woman, and circulate to the rest of the OptiBreech team. Any cases in which a potential increased risk has been identified must be reviewed by the Breech Lead Obstetrician. The team member will also make a plan for any further follow-up that needs to occur antenatally, concerning breech presentation. From this point, the woman should be caseloaded by the OptiBreech team, unless she is already booked with a caseload team. In this case, the OptiBreech team should offer support to the caseload midwife and work in collaboration with them.

In addition to the Participant Information Sheet, women randomised to OptiBreech care will be given the OptiBreech Care Information Sheet and offered an opportunity to discuss and ask questions. This explains the intervention they have been randomised to, why we are researching this care pathway, and what her options are. It provides information about mode of birth as provided in the RCOG guideline but also includes information about the uncertainties which the study aims to address. This information sheet was developed with extensive input from the PPI group to ensure it was understandable and clear about the potential risks and benefits of OptiBreech care.

Women randomised to the standard care pathway should receive the RCOG ‘Breech Baby at the End of Pregnancy’ leaflet, <https://www.rcog.org.uk/en/patients/patient-leaflets/breech-baby-at-the-end-of-pregnancy/>. This explains standard care, in addition to any standard leaflets included in local guidelines. Women should be offered an opportunity to discuss and ask questions before decided on whether to accept the offer of ECV or explore another option.

A counselling pro forma is also included in the hand-held CRF packet, to help ensure balanced, consistent, evidence-based counselling. It is based on the governance-approved pro forma included in the Trust guideline for the lead site. It can be used for women randomised to either arm of the trial.

## Autonomy and individualised care

This pilot trial is designed so that women randomised to both standard care and to OptiBreech care retain their autonomy. As in standard care, if the woman prefers not to have an ECV or prefers not to plan a VBB with OptiBreech care, this should be respected. This will also enable us to determine how acceptable each intervention is based on its acceptance and attrition rates. The interventions we aim to trial therefore reflect the reality of contemporary maternity care, where the preferred or most favourable option is recommended, but the woman’s autonomy should be respected. However, in the context of breech presentation, autonomy itself is affected by a number of factors. Although a woman can opt out, can change her mind and can make a different choice, in reality these choices may be limited by how near to giving birth she is and the availability of different methods near where she lives. While a woman may choose, this choice may be influenced by the experience, skills and attitudes of her caregivers. A pragmatic, multi-site trial design will help us to observe the effectiveness of these care pathways in the context of this inevitable variation, despite our best efforts to limit variation as much as possible.

## Attendance in labour and clinical responsibility

Each site should establish local arrangements for how the OptiBreech team will be contacted when a woman receiving OptiBreech care is in labour. In all cases, a core or caseload midwife should be assigned to provide care under the guidance of the OptiBreech team member.

The OptiBreech team should be involved from the beginning of labour assessments. This may not be in person in early labour, but the OptiBreech team member will be considered the clinical lead. Any clinical circumstances suggesting the need for intervention or a change in management should be reviewed and agreed with the OptiBreech team member, in collaboration with the on-call obstetric team. The OptiBreech team member supervising the birth should be present in the labour room throughout all of the second stage of labour at a minimum.

Clinical care, including facilitating a straightforward breech birth, should be provided by a midwife on duty who has completed the OptiBreech training. They need not yet be considered fully proficient as long as the birth is supervised by the OptiBreech team member. The clinical team may also decide, with consent from the birthing person, that the birth will be attended by an on-call obstetrician, for training purposes, including upskilling obstetric staff to provide care as part of the OptiBreech team. It is entirely appropriate for training to continue as usual in participating sites. However, the fully proficient OptiBreech team member must remain present and in the role of clinical lead supervising the birth, to ensure the fidelity of the intervention.

The OptiBreech team members should communicate clearly and often with the clinical team on duty, especially if difficulty is anticipated. The on-duty consultant obstetrician or senior obstetric registrar is encouraged but not required to be in the room at the time of the birth, in order to facilitate closer teamwork in case complications or difficulties arise. If the OptiBreech team member feels that either forceps (for midwives) or a CS is necessary, the OptiBreech team member should clearly state their recommendation to the woman and the senior obstetrician on duty. They should also state clearly to both parties that they are handing over care and document this in the notes. They may stay to assist the clinical team if all agree this is helpful, but it is not required. In cases where the OptiBreech lead has recommended a CS and the woman or other staff decline, this is recorded in the CRF.

OptiBreech clinical responsibility will finish when care is handed over or the birth is complete, with the birthing person and neonate stabilised. On-duty labour ward staff should provide all standard follow-up care, including suturing, unless otherwise agreed.

## Maternal birthing posture

OptiBreech team members should be confident to support women to give birth in the position of their choice, including upright positions, e.g. kneeling, hands/knees, on a birthing stool, standing/squatting. This is consistent with current RCOG guidelines^16^ but may vary from local guidelines. Robustly evaluated PBB training^48,53^ provides professionals with skills to resolve obstructions when women give birth in upright positions. All OptiBreech team members will have this training and be assessed in the performance of upright manoeuvres in simulation by the local PI.

## Use of algorithm

Evaluated PBB training is summarised in the Physiological Breech Birth Algorithm. OptiBreech team members should refer to this algorithm for guidance during training. Two elements in the algorithm are included in the assessment of intervention fidelity:

Maternal movement and effort: After the breech remains visible on the perineum between contractions, following any delay >90 seconds, the clinician should encourage maternal movement (if upright) and effort (pushing). Hands-on interventions should be applied only after this is ineffective. This is a cornerstone of PBB practice and represents a significant change in practice for most professionals.^69^

*Pelvis-to-head interval:* The fetal head should be delivered no longer than 5 minutes after the birth of the pelvis. This includes time to perform manoeuvres. Evidence from video analysis indicates that the interval between birth of the pelvis and birth of the head is significantly shorter in PBBs than the intervention thresholds recommended in current RCOG guidelines,^16^ so this represents a somewhat stricter timeframe for delivery than the current guidelines.

## Recordkeeping

All breech care is documented in the CRF. This includes the Physiological Breech Birth Documentation Pro Forma, which should be used to document timings around the time of birth.

# Assessment of safety

Serious adverse events are expected to occur in maternity care, although at a low rate in this cohort. The RCOG guideline^16^ reports a perinatal death rate of 0.5/1000 for caesarean delivery and 2/1000 for vaginal breech birth. Admissions to neonatal care also occur following all modes of delivery at a rate of about 5-7% at term.

The research sponsor, King’s College London, has a responsibility to ensure the safety of research participants. The local PI has responsibility for ensuring all SAEs are reported. The CI also has co-ordinating responsibility for reporting adverse events to the Trial Steering Committee, the Sponsor, the Research and Development Office (R&D) and to the relevant Research Ethics Committee (REC), and for the submission of an Annual Safety Report.

## Ethics Reporting

Reports of related and unexpected SAEs will be submitted to the Main REC within 15 days of the CI becoming aware of the event, using the NRES template. A copy of the SAE notification and acknowledgement receipt will be sent to the R&D Directorate.

Maternal or neonatal deaths, and admissions to the NICU for longer than 4 days should be reported immediately to the CI as SAEs.

The following neonatal SAEs are expected and should be recorded and reported according to the protocol:

| Admission to the NICU for up to 4 days  Low Apgar score  Low cord blood gases  Neonatal resuscitation  Hematoma  Haemorrhage  Spinal cord injury  Skull fracture  Bone fracture  Peripheral nerve injury/Brachial plexus injury present at discharge from hospital  Facial nerve paresis  Significant genital injury  Laceration to baby buttocks  Respiratory distress syndrome requiring treatment  Neonatal seizures or convulsions  Neonatal encephalopathy  Necrotizing enterocolitis  Perinatal infection  Neonatal hypoglycemia requiring treatment  Hyperbilirubinemia / neonatal jaundice requiring treatment  Stupor/decreased response to pain/coma  Facial palsy |
| --- |

The following maternal SAEs are expected and should be recorded and reported according to the protocol:

Haemorrhage

Obstetric Anal Sphincter Injury (OASI)

Admission to higher-level care for up to 4 days

Cervical laceration involving lower uterine segment

Vertical uterine incision or serious extension to transverse uterine incision

Bladder, ureter or bowel injury requiring repair

Dilation & curettage for bleeding or retained placental tissue

Manual removal of placenta

Uterine Rupture

Hysterectomy

Vulval or perineal haematoma requiring evacuation

Wound dehiscence / breakdown

Wound infection requiring prolonged hospital stay / readmission / antibiotics

Sepsis

Disseminated Intravascular Coagulation

Re-admission within 28 days following birth

Table 4: Information with regards to Safety Reporting

|  | **Who** | **When** | **How** | **To Whom** |
| --- | --- | --- | --- | --- |
| **SAE** | Chief Investigator | -Report to Sponsor within 24 hours of learning of the event  -Report to the MREC within 15 days of learning of the event  - Report to TSC within 15 days of learning of the event | SAE Report form for Non-CTIMPs, available from NRES website. | Sponsor, MREC and TSC |
| **Urgent Safety Measures** | Chief Investigator | Contact the Sponsor and MREC Immediately  Within 3 days | By phone  Substantial amendment form giving notice in writing setting out the reasons for the urgent safety measures and the plan for future action. | Main REC and Sponsor  Main REC with a copy also sent to the sponsor. The MREC will acknowledge this within 30 days of receipt. |
| **Progress Reports** | Chief Investigator | Annually ( starting 12 months after the date of favourable opinion) | Annual Progress Report Form (non-CTIMPs) available from the NRES website | Main REC |
| **Declaration of the conclusion or early termination of the study** | Chief Investigator | Within 90 days (conclusion)  Within 15 days (early termination)  *The end of study should be defined in the protocol* | End of Study Declaration form available from the NRES website | Main REC with a copy to be sent to the sponsor |
| **Summary of final Report** | Chief Investigator | Within one year of conclusion of the Research | No Standard Format  However, the following Information should be included:-  Where the study has met its objectives, the main findings and arrangements for publication or dissemination including feedback to participants | Main REC with a copy to be sent to the sponsor |

## Trial Steering Committee

A Trial Steering Committee will be appointed, whose role it is to oversee the project and to make a recommendation regarding feasibility of a substantive RCT, based on the results of the feasibility work. The TSC can call an ad hoc meeting at any time to discuss concerns arising from safety reporting. More detail is outlined below, under Study Oversight.

## Ethics and regulatory approvals

As this proposed research project will be conducted within the NHS in England, permission will need to be sought through the Health Research Authority (HRA). The project will be conducted in accordance with the principles of Good Clinical Practice (GCP). A favourable ethical opinion will be sought form the appropriate REC and local Research and Development approvals obtained prior to commencement of the study.

Detailed discussion of ethical considerations is given below.

# Compliance and withdrawal

## Subject compliance

This is a pragmatic trial comparing two care pathways. Rather than directly compare ECV with OptiBreech Care for a vaginal breech birth without an ECV attempt, we aim to compare a care pathway that offers one or the other as the first-line intervention. This is because the effectiveness of a care pathway depends on more than the efficacy of the intervention. Its acceptability to women and interactions with other components of the care pathway affect its clinical and cost effectiveness.

It is therefore expected that a certain percentage of women randomised to standard care will choose to plan a CS or a VBB, and a certain percentage of women randomised to OptiBreech Care will choose to attempt an ECV or plan a CS (See Figure 4: Flow of participants through OptiBreech Care trial). These acceptance rates will be evaluated as part of this study and be included in feasibility calculations.

## Dropout of participants

Women may withdraw at any time without giving a reason, but once their information has been anonymised, we will keep the anonymised data. This is explained in the Participant Information Sheet. We will mark their database entry as ‘withdrawn,’ along with the date. They will receive no further contact, and we will seek to obtain no further information about them.

Where a participant decides to withdraw the use of their data before data about the birth outcome has been collected, this participant will be replaced by another randomised participant.

Women can choose not to participate in follow-up surveys, even when they have already consented to participate. Where people have given consent for use of their data but have been lost to follow-up, this will be recorded as incomplete data. An aim of the feasibility work is to evaluate the completeness of the data collection process.

## Protocol compliance

Protocol compliance will be monitored as part of the evaluation of fidelity to the intervention. The three key fidelity criteria – presence of an OptiBreech team member at vaginal breech births, use of maternal movement and effort prior to hands-on intervention, and <5 minutes from birth of pelvis to completion – are recorded on the CRF. A report on fidelity to intervention will be made to the TSC during its review at the end of the randomisation pilot.

Our experience in OptiBreech 1 has been that PIs have contacted the CI to discuss known issues with protocol compliance. Some of these have been formally discussed through the qualitative interviews as part of the implementation evaluation. Others have been discussed during on-line webinars conducted to support on-going training, reflection and learning in OptiBreech sites. These challenges have been resolved by discussions with the PI and local maternity team leaders. We will maintain regular communication with all PIs in the OptiBreech Care trial and encourage a similar problem-solving approach.

Cases of recurrent non-compliance will first be discussed with the local PI. As this is a feasibility study, it is important to identify potential issues that may affect the site’s ability to deliver the intervention with fidelity. Cases where non-compliance is significant or may potentially put women at increased risk, eg. planned, non-urgent vaginal breech births where no one meeting the proficiency criteria was in attendance, or where they were not permitted to attend by the staff on duty, may result in stopping recruitment within that site.

# Data

## Data to be collected

The data we aim to collect is influenced by the following factors:

1. Our systematic review of outcomes reported in effectiveness studies of breech birth at term;^54^
2. The views of our PPI group members about what outcomes are important to women but under-reported or not reported at all;^54^
3. The need to pilot data collection on all endpoints that may be used in a substantive trial; and
4. Our intention to determine and eventually compare the factors that impact cost effectiveness in the control and intervention pathways.

We have listed all dates and times as ‘date’ in data type, although our intention is to calculate time-to-event intervals where relevant. Depending on the woman’s care pathway and personal choices, not all categories of data will require completion (e.g. ECV, induction, labour care). Additionally, almost all of the data points would be routinely collected during thorough contemporaneous documentation. We have outlined the data we need in order to understand how these variables interact within the care pathways.

| Baseline data | | | | | |
| --- | --- | --- | --- | --- | --- |
|  | Source  N = electronic or handheld record  P = participant  A = procedure | Why  * = explanatory | Standardised tool or procedure | Form of data | |
| Estimated date of birth | A | * | USS or patient-reported dates | date | |
| By menstrual dates or ultrasound | N | explanatory |  | binary | |
| Point of diagnosis | N | eligibility |  | binary | |
| Referred by? | N | * |  | categorical | |
| Name | P | Follow-up |  |  | |
| Date of Birth | P | * |  | date | |
| Contact details | P | Follow-up |  |  | |
| NHS Number and Medical Record Number | N | Follow-up |  | ID | |
| PAS General Practitioner ID | N | Follow-up |  | ID | |
| Post Code | P | * |  |  | |
| Gender | P | * |  | categorical | |
| Ethnicity | P | * |  | categorical | |
| First language spoken | P | * |  | categorical | |
| Interpreter required? | P |  |  |  |  |
| Highest level of education | P | * |  | categorical | |
| Baby feeding plans | P | * |  | categorical | |
| Planned place of birth prior to diagnosis | P | * |  | categorical | |
| Parity | N | * |  | categorical | |
| Height and Weight at booking | A | * | Routine | continuous | |
| Rhesus status | A | * | Routine blood test | binary | |
| Previous pregnancy complications | N | * |  | categorical | |
| Maternal concerns, including uterine anomalies | N | * |  | categorical | |
| Fetal concerns | N | * |  | categorical | |

| During treatment | | | | |
| --- | --- | --- | --- | --- |
|  | Source  N = electronic or handheld record  P = participant  A = procedure | Why  * = explanatory | Standardised tool or procedure | Form of data |
| Date of counselling | N | * |  | date |
| Role & training of person counselling | N | * |  | categorical |
| Initial plan following counselling | N | outcome |  | categorical |
| Number of antenatal appointments | N | economic |  | ordinal |
| Role of professional at antenatal appointments | N | economic |  | binary |
| Ultrasound scans | A | economic | Standardised procedure indicated by care needs |  |
| Date | N | * |  | date |
| Performed by | N | economic |  | categorical |
| Purpose | N | * |  | categorical |
| Type of breech presentation | A | * |  | categorical |
| Hyper-extended fetal head? | A | * |  | binary |
| Nuchal cord visualised | A | * |  | binary |
| Estimated fetal weight | A | * |  | ordinal |
| Fetal growth centile | A | * |  | ordinal |
| Growth trajectory | A | * |  | binary |
| Head circumference | A | * |  | ordinal |
| Femur length | A | * |  | ordinal |
| Abdominal circumference | A | * |  | ordinal |
| Amniotic Fluid Index | A | * |  | ordinal |
| Single deepest pool | A | * |  | ordinal |
| Methods of encouraging the baby to turn head-down | | | | |
| Did the person receive counselling on methods? | N | * |  | categorical |
| Additional items given concerning how to turn the baby | N | Economic |  | categorical |
| External cephalic version | | | | |
| Total number of ECV attempts | A | Outcome and economic | Routine / chosen procedure | ordinal |
| Date | N | * |  | date |
| Location | N | * |  | categorical |
| Professional performing | N | * |  | categorical |
| Experience level of operator | N | * |  | categorical |
| One operator or two | N | * |  | binary |
| Abdominal lubricant | N | * |  | categorical |
| Tocolytic used, dose and route | A | * | Drug administration | categorical |
| Analgesia/anaesthetic used | A | * | Drug administration | categorical |
| Number of attempts on this date | N | * |  | categorical |
| Total hours admitted for procedure | N | Economic |  | ordinal |
| Anti-D administered? | A | Economic | Drug administration | binary |
| Inpatient admission | N | Outcome and economic |  | ordinal |
| Emergency delivery required | N | Outcome |  | binary |
| Successful? | N | Outcome |  | binary |
| Requesting a 2^nd^ attempt | N | * |  | binary |
| Planned mode of birth following this ECV attempt | N | * |  | categorical |
| Induction of Labour | | | | |
| Number of cervical sweeps | A | * | Routine / chosen procedure | ordinal |
| Date | N | * |  | date |
| Other methods of induction used | N | * |  | categorical |
| Date/time admitted for induction of labour | N | * |  | date |
| Methods used | A | * | Routine / chosen procedure | categorical |
| Labour Care | | | | |
| Date/time admitted | N | * |  | date |
| Initial place of care | N | * |  | categorical |
| Vaginal examination – dates and time | A | * | Routine / chosen procedure | date |
| Dilatation and station | N | * |  | ordinal |
| Type of fetal monitoring used in first stage | A | * | Routine / chosen procedure | categorical |
| Meconium-stained liquor in first stage of labour | N | * |  | binary |
| Oxytocin infusion started AFTER the onset of active labour? | A | * | Drug administration | binary |
| Time | N | * |  | date |
| Was oxytocin infusion started AFTER the onset of active labour | N | * |  | binary |
| Was an amniotomy performed AFTER the onset of active labour? | A | * | Routine / chosen procedure | binary |
| Analgesia / Anaesthetic | A | * | Drug administration | categorical |
| Date/time second stage of labour started | N | * |  | date |
| Date of start of expulsive pushing effort | N | * |  | date |
| Type of fetal monitoring used for second stage | A | * | Routine / chosen procedure | categorical |
| Maternal birthing position | N | * |  | categorical |
| Lead attendant | N | * |  | categorical |
| Continuity of carer | N | outcome |  | binary |
| Cord prolapse | N | * |  | categorical, date |
| Placental abruption | N | * |  | Categorical, date |
| Time of onset of spontaneous respirations | N | * |  | date |
| Vaginal breech births only | | | | |
| Experience level of lead attendant | N | * |  | categorical |
| Had professional attended OptiBreech training? | N | * |  | binary |
| Professional who met OptiBreech proficiency criteria? | N | * |  | binary |
| Presenting part first seen | N | * |  | date |
| Time first visible | N | * |  | date |
| Anterior buttock first visible | N | * |  | date |
| Was the birth filmed | N | * |  | binary |
| Maternal position at start of emergence | N | * |  | categorical |
| Both buttocks/anus visible on perineum between contractions | N | fidelity |  | date |
| Position of fetal pelvis at emergence | N | * |  | categorical |
| Pelvis born | N | * |  | date |
| Umbilicus born | N | * |  | date |
| Nipple line / scapulae visible | N | * |  | date |
| Legs born | N | * |  | date |
| Arms born | N | * |  | date |
| Head born | N | fidelity |  | date |
| Umbilical cord wrapping | N | * |  | categorical |
| Encourage maternal movement and effort | N | fidelity |  | binary |
| Episiotomy | N | * |  | binary |
| Change maternal position | N | * |  | binary |
| Time | N | * |  | date |
| Fundal pressure | N | * |  | binary |
| Assistance applied | N | * |  | date |
| Interventions used | N | * |  | categorical |
| Birth outcomes | | | | |
| Date/Time of birth | N | * |  | date |
| Place of birth | N | * |  | categorical |
| Mode of birth | N | outcome |  | categorical |
| Maternal birth position | N | * |  | categorical |
| Numbers of staff present for the birth | N | Economic |  | categorical |
| Time cord was cut | N | * |  | date |
| Skin-to-skin immediately following birth | N | outcome |  | binary |
| Length of time | N | outcome |  | ordinal |
| Date/time | N | * |  | date |
| If CS, Category | N | outcome |  | categorical |
| If CS, reason | N | * |  | categorical |
| Dilation at CS | N | outcome |  | ordinal |
| Station at CS | N | outcome |  | ordinal |
| Fetal pillow used to assist elevation? | N | * |  | categorical |
|  |  |  |  |  |
| Infant Feeding | | | | |
| Initiated breastfeeding? | N | outcome |  | binary |
| Method of feeding on discharge from labour care/hospital | N | outcome |  | binary |
| Method of feeding on discharge from care | N | outcome |  | categorical |
| Maternal outcomes | | | | |
| Maternal death prior to discharge from maternity care | N | outcome |  | binary |
| Date | N | * |  | Date |
| Cause of death | N | * |  | Categorical |
| Estimated blood loss | N | outcome |  | Continuous |
| Transfusion received? | A | outcome | Blood products | Binary |
| Anemia requiring treatment | A | outcome | Drug administration | binary |
| perineum | N | outcome |  | categorical |
| Degree | N | outcome |  | ordinal |
| Admission to higher-level care | N | Outcome |  | Binary |
| Total inpatient nights | N | economic |  | continuous |
| Other trauma or morbidity | N | outcome |  | binary |
| Readmission within 28 days | N | Outcome |  | binary |
| Total number of postnatal midwifery visits | N | Economic |  | continuous |
| Baby Outcomes | | | | |
| Name | N | Follow-up |  | ID |
| NHS Number and Medical Record Number | N | Follow-up |  | ID |
| Sex | N | * |  | categorical |
| Birth weight | N | * | Standardised procedures | continuous |
| Gap centile | N | * | Standardised tool | continuous |
| Head circumference | N | * | Standardised procedure | continuous |
| Apgar, 1 minute and 5 minutes | N | Outcome | Standardised tool | ordinal |
| Live birth / stillbirth / neonatal death | N | Outcome |  | categorical |
| Date | N | * |  | date |
| Cord blood gases | N | outcome | Standardised tool | continuous |
| Resuscitation required | N | outcome |  | binary |
| Was resuscitation initiated with the umbilicus intact? | N | outcome |  | binary |
| Admission to neonatal unit / special care baby unit / transitional care | N | outcome |  | binary |
| Reason | N | * |  | Categorical |
| Number of nights | N | Economic |  | Continuous |
| Severe morbidity | N | Outcome |  | binary |
| Additional trauma or morbidity | N | Outcome |  | binary |
| Randomised participants only | | | | |
| How much time did someone spend on-call to support this birth? | Staff reported | Economic |  | continuous |
| At any point during labour, did the lead attendant (OptiBreech team member if present) advise a caesarean birth? | N | fidelity |  | binary |
| Was the birth presented to others for teaching purposes, including simulation if appropriate? | Staff reported | fidelity |  | binary |

| Follow-up | | | | |
| --- | --- | --- | --- | --- |
| Survey 1 month following birth | | | | |
|  | Source  P = patient-reported | Why  * = explanatory | Standardised tool or procedure | Form of data |
| I got the information that was relevant to me | P | outcome | 5-point Likert scale based on validated instrument^70^ | continuous |
| I was offered little choice about my care | P | outcome |  | binary |
| I felt I was treated as an individual | P | outcome |  | binary |
| I could discuss what was important to me | P | outcome |  |  |
| I was overwhelmed with the information | P | outcome |  |  |
| The extra care I received made me feel that my baby was safe | P | outcome |  |  |
| The worry nearly became too much for me | P | outcome |  |  |
| When you were pregnant, did you use any of the following to encourage your baby to turn head-down? | P | * |  | categorical |
| Did you have an attempt at turning the baby to head-down by a doctor or midwife, also known as ECV or external cephalic version? | P | * |  | binary |
| If you were pregnant with a breech baby again, would you choose to have an attempt at turning the baby (ECV)? | P | Outcome |  | binary |
| If you had an attempt at turning the baby, how painful did you find the procedure to be? | P | Outcome | Visual analogue scale | continuous |
| Childbirth experience questionnaire | P | Outcome | 22 items, validated for use in the UK^59,60^ |  |
| Did someone present at the birth speak to you afterwards, to help you understand what happened during the birth? | P | Outcome |  | categorical |
| Readmission to hospital | P | outcome |  | categorical |
| Time spent in hospital | P | Economic |  | continuous |
| Separation from baby | P | Outcome |  | categorical |
| Visiting the GP | P | Outcome |  | categorical |
| Number of visits | P | Economic |  | continuous |
| Health-related quality of life | P |  | PROMIS-10^61^ |  |

| Follow-up | | | | |
| --- | --- | --- | --- | --- |
| Survey 3-4 month following birth | | | | |
|  | Source  P = patient-reported | Why  * = explanatory | Standardised tool or procedure | Form of data |
| Use of formal ‘Birth Reflections’ service | P | Outcome |  | binary |
| Readmission to hospital since last survey | P | outcome |  | binary |
| Time spent in hospital | P | Economic |  | continuous |
| Visiting the GP | P | Outcome |  | binary |
| Number of visits | P | Economic |  | continuous |
| Urinary incontinence | P | Outcome | 4-point scale used in previous breech trials^45^ |  |
| Fecal incontinence | P | Outcome |  |  |
| Incontinence of flatus | P | Outcome |  |  |
| Depression or anxiety requiring treatment | P | Outcome |  | binary |
| Frequent distressing memories or dreams about pregnancy and/or birth experience | P | Outcome |  | binary |
| Health-related quality of life | P | economic | PROMIS-10^61^ |  |
| Method of baby feeding | P | Outcome |  | Categorical |
| Has baby had a hip scan? | P | Outcome |  | binary |
| Has baby been admitted to hospital? | P | Outcome |  | Binary |
| How much time? | P | Economic |  | Continuous |
| Baby seen the GP? | P | Outcome |  | Binary |
| How many times? | P | economic |  | continuous |
| Ages and Stages Questionnaire | P | Outcome & economic | Standardised tool used at 3-4 months^62^ |  |

| Follow-up | | | | |
| --- | --- | --- | --- | --- |
| Survey 2 years following birth | | | | |
|  | Source  P = patient-reported | Why  * = explanatory | Standardised tool or procedure | Form of data |
| Readmission to hospital since last survey | P | outcome |  | binary |
| Time spent in hospital | P | Economic |  | continuous |
| Visiting the GP | P | Outcome |  | binary |
| Number of visits | P | Economic |  | continuous |
| Urinary incontinence | P | Outcome | 4-point scale used in previous breech trials |  |
| Fecal incontinence | P | Outcome |  |  |
| Incontinence of flatus | P | Outcome |  |  |
| Depression or anxiety requiring treatment | P | Outcome |  | binary |
| Frequent distressing memories or dreams about pregnancy and/or birth experience | P | Outcome |  | binary |
| Health-related quality of life | P | economic | PROMIS-10^61^ |  |
| Method of baby feeding | P | Outcome |  | Categorical |
| Has baby been admitted to hospital? | P | Outcome |  | Binary |
| How much time? | P | Economic |  | Continuous |
| Baby seen the GP? | P | Outcome |  | Binary |
| How many times? | P | economic |  | continuous |
| Ages and Stages Questionnaire | P | Outcome & economic | Standardised tool used at 1 year of age^62^ |  |
| Have you experienced another pregnancy within the past year? | P | outcome |  | binary |
| Miscarriage? Date | P | outcome |  | date |
| Termination? Date | P | Outcome |  | date |
| Has this baby been born yet? | P | * |  | binary |
| Where did you are or you planning to give birth? | P | Follow-up |  |  |
| (no) What is the expected date of birth? | P |  |  | date |
| (yes) Actual date of birth | P |  |  | date |
| Was this baby in breech presentation after 36 weeks? | P | Outcome |  | binary |
| Mode of birth | P | Outcome |  | categorical |
| Is there anything you would like to tell us about how your breech pregnancy has affected this pregnancy? | P |  |  | open |
| May we have your consent to access records about this birth in order to record the outcomes? This is to understand more about what happens in future pregnancies after a breech presentation in pregnancy. We will not record identifiable details about this baby. | P | consent |  | binary |
| Subsequent pregnancies – entered by research staff | | | | |
| Date of birth | N | * |  | date |
| Breech presentation after 36 weeks? | N | * |  | binary |
| Immediate neonatal outcome | N | Outcome |  | categorical |
| (If stillbirth or neonatal death) Date of death: | N | * |  | date |
| Neonatal severe adverse outcome | N | outcome |  | binary |
| Maternal mortality? | N | outcome |  | binary |
| (if death) date | N | * |  | date |
| cause of death | N | * |  |  |
| Maternal severe adverse outcome | N | outcome |  | binary |

## Data quality and validity

We will conduct at least one site visit with each participating site. The purpose of this visit will be to provide training, review the site file and check the data in a sample CRF against anonymised clinical notes. Where discrepancies are noted, we will provide support to the site and return if necessary. A record of these visits and findings will be kept. We will also use them to develop and pilot an audit form to identify potential issues that should be checked during any future substantive trial.

## Data handling and record keeping

The local PI is responsible for data collection, recording and quality, unless otherwise delegated to a member of the local research team and recorded on the delegation log. All investigators and study site staff involved with this study must comply with the requirements of the Data Protection Act 2018 with regard to the collection, storage, processing and disclosure of personal information and will uphold the Act’s core principles.

Data will be collected via an eCRF directly onto a database maintained by MedSciNet, using a secure login and password. Paper-based CRF forms will be provided to facilitate data collection prior to entry, where this is preferrable for sites or for participants. Anonymised data will be downloaded from the database at the end of the study period.

All data, and scanned copies of paper-based CRFs where these have been used, these should be submitted to the research team within 6 weeks of the birth, using either an encrypted NHS e-mail transfer (Shawn.Walker1@nhs.net) or the Trust’s secure file transfer system. Here it will be kept on a secure computer file on the KCL secure online storage network. Computers used to collate and analyse the data will have limited access measures via user names and passwords. Study sites will keep their hard files for the usual duration after the end of study at their site.

Published results will not contain any personal data that could allow identification of individual participants.

As this is a study involving pregnant women and research records should be retained according to NHS Guidelines for the retention of documentation involving pregnant women. All medical records will be retained for at least 25 years after publication of the final study report. We plan to retain all research data for 10 years, because this is the potential follow-up timeframe stipulated on the consent form.

Following the end of the study, anonymised data will be archived on the university’s secure Sharepoint site. This will be accessible only to the research team.

Anonymised data will be stored in a Microsoft Excel Spreadsheet. All person-identifiable information will be removed or altered. For example, all dates will be converted to time-to-event intervals from the estimated date of birth, or actual data of birth for follow-up surveys. Prospective consent is obtained to use anonymised data in future studies, subject to appropriate ethics and data access approvals.

## Anonymisation of data

Confidentiality will be maintained by use of a Patient Details Database, which is a separate, but linked database. Each participant will be allocated a study code, which will link the two datasets, in case patient details are required for future contact as consented or for data queries. When participant identifiers (first name, last name, date of birth, hospital number, NHS number, etc.) are entered into the MedSciNet database at enrolment (for woman/birthing person), or following the birth (for baby), this information will be automatically and immediately transferred from the main database to the Patient Details Database. In this database, personal details will be stored separately and encrypted, for GCPR-compliant security.

Access to the Patient Details Database will be by separate login. While each local Data Collection Centre will have access to its own participants’ personal data, only the Project Lead and Project Manager will have access to the main file. No personal details will be transferred to a third party for any reason.

# Statistical considerations

The sample size calculation for this pilot trial was carried out by the CI, Dr Shawn Walker, under the supervision of Dr Kirsty Logan, Senior Clinical Research Epidemiologist at King’s Health Partners Institute of Women and Children’s Health.

## Sample size calculation

The sample size of 104 women was calculated to enable estimation of a recruitment rate between 20-80%, with a 95% confidence interval, within $\pm$10%.

We expect the pilot trial to last 6 months, in 5 NHS sites, with a minimum of 10 recruits per site. In the five months after the first OptiBreech 1 site opened, over 22 women were recruited who wished to plan a vaginal breech birth. This is from a much smaller recruitment pool (only women who know they would like to plan a vaginal breech birth) than the OptiBreech Care Trial, in which all eligible women are invited to participate.

## Statistical analysis

Feasibility data will be analysed using descriptive statistics. All data analysis will be undertaken blind to allocation. The following will be reported, using the data obtained from the OptiBreech mTC Database:

- Recruitment rate recorded as the number of eligible participants who consent to participate in the study by 6 months (randomised) and overall (non-randomised);
- Acceptance rate recorded as the number of participants randomised to OptiBreech Care who plan a vaginal breech birth, and the number of participants randomised to the control who attempt an ECV;
- Attrition rate recorded as the number of participants who consent to participate who remain in the study until the end of follow-up at 4 months after birth;
- Long-term attrition rate recorded as the number of OptiBreech 1 participants who complete 1-year and 2-year follow-up surveys when invited;
- Fidelity to intervention recorded as number of planned VBBs attended by a proficient team member;
- Costs to deliver the service recorded as total number of days and nights spent on call to support planned VBBs in the trial by 6 months;
- Neonatal admission rates according to intention to treat in the trial and actual mode of birth;
- Mode of birth by intention to treat (care pathway) and intended mode of birth;
- All severe adverse neonatal and maternal outcomes by intention to treat, intended mode of birth and actual mode of birth.

In a future trial ‘intention to treat’ will make reference to the flow of women/birthing people through the breech care pathway as depicted in Figure 4 below.

Figure 4: Flow of women/birthing people through the breech care pathway

Potential primary outcomes for a trial could be:

**Randomised to ‘offer ECV’ vs ‘offer OptiBreech care’ (orange)**

1. Potential primary outcome = neonatal admission. In the latest Cochrane Review,^37^ neonatal admission occurs 121/1000 following ECV.
2. Potential secondary outcome = Mode of birth. In the latest Cochrane Review,^37^ CS rate is 180/1000 following ECV.

**Cohort analysis**

Potential primary outcome = composite perinatal death or serious morbidity (maternal and neonatal) following enrolment

1. Groups 1: ECV (grey) versus planned VBB with no ECV (dark green). Cochrane review: Incidence of Apgar <7 at 5 minutes, 44/1000 following ECV, 70/1000 following no ECV, considered equivalent.
2. Groups 2: VBB with OptiBreech care (planned and unplanned) versus VBB without OptiBreech care (planned and unplanned) (all green, subgroups). In the Term Breech Trial,^45^ this rate was 5%. In the PREMODA cohort study,^71^ it was 1.6%.
3. Additional comparisons: all planned CS (all blue) vs all planned cephalic birth after ECV (yellow) vs VBB with OptiBreech care vs VBB without OptiBreech care (all green)
   1. Planned cephalic birth after ECV as control

Potential secondary outcome = mode of birth

1. Comparison: ECV attempted (grey) versus planned VBB with no ECV (dark green)
2. Comparison: VBB with OptiBreech team versus no OptiBreech team present (all green, subgroups)

We will report on incidence rates for all of these outcomes that may be used as comparators.

## 14.3 Interim analysis and data monitoring

### Green/Amber/Red Criteria for recommending a full RCT

As this is a pilot trial, there will be no interim analysis. Rather, the TSC review at the end of the pilot trial will serve as data monitoring, as part of the overall decision about whether to proceed with a substantive trial. All unexpected serious adverse events will be reported to the TSC, and the TSC will have the power to pause the trial to assess unblinded data if that appears necessary for ensuring participants’ safety, as well as the power to stop the trial if participants’ safety appears to be at risk.

The following will be used to determine the feasibility of comparing standard care versus OptiBreech care:

A = total number of women randomised to OptiBreech VBB care

B = number of women declining OptiBreech VBB care in favour of ECV or CS first

C = total number of women randomised to ECV / standard care

D = number of women declining ECV attempt in favour of VBB or CS first

E = number of women lost to follow-up

F = number of sites participating in OptiBreech 2

G = number of months to achieve 104 recruits

H = number of sites in OptiBreech 1 meeting requirements for participation

J = number of additional sites indicating interest in joining the trial

Total women required for randomisation: 2280 to compare the outcome of ‘neonatal admission’ using current evidence on incident rates

48 women / month for 48 months

Green for trial: ($\frac{\left( A-B \right)+\left( C-D \right)-E}{F(G)})*(F+H)$ > 48 women

Amber: ($\frac{\left( A-B \right)+\left( C-D \right)-E}{F(G)})*(F+H+J)$ > 48 women

Red: ($\frac{\left( A-B \right)+\left( C-D \right)-E}{F(G)})*(F+H+J)$ < 48 women

For example:

104 women (A+C) are recruited by 5 months (G = 5)

52 are randomised to OptiBreech care (A = 52) but 30 decline this in favour of ECV or CS (B = 30)

52 are randomised to ECV (C = 52) but 20 decline this in favour of CS or VBB (D = 20)

2 women are lost to follow-up (E = 2)

5 sites participated in OptiBreech 2 (F = 5)

10 more sites in OptiBreech 1 are meeting participating criteria (H = 10)

25 more sites not currently participating have expressed an interest (J = 25)

($\frac{\left( A-B \right)+\left( C-D \right)-E}{F(G)})*(F+H)$ = 31.2

($\frac{\left( A-B \right)+\left( C-D \right)-E}{F(G)})*(F+H+J)$ = 83.2

The feasibility trial will meet the Amber light criteria. Depending on the number of sites who are able to develop their service enough to meet the criteria, it should be feasible to compare standard care with OptiBreech VBB care in a randomised trial delivered within 48 months.

### Monitoring, quality control and assurance

This feasibility study is being completed as part of an NIHR Advanced Fellowship. All statistical calculations will be done by the Fellow and Chief Investigator, Dr Shawn Walker, who will have access to statistical support within the research team. An independent statistician will be appointed to the TSC, and they will be provided with all original data.

Local R&D offices will ensure all PIs have current good clinical practice training in place before authorising the site to open.

# Ethical considerations

## COVID-19 precautions

This research is being initiated during a pandemic and associated public health social distancing measures to limit the spread of the virus. All hospital and government policies will be followed to maintain these precautions, for as long as they are in effect.

Wherever possible, we will seek to provide participant information and to take consent electronically, to minimise contact. Where patients have not provided an e-mail address to the NHS service providing their maternity care, or not given permission for it to be used in this way, we will provide a paper copy. Any PIS or consent form will be made available for download for all participants.

We will also record a video description of the study and explanation of the consent form, provided exactly as it would be in practice, to minimise face-to-face time required to take consent. We will also record a description of physiological breech birth and the informed decision-making content available from the current RCOG guideline, to minimise face-to-face contact time and ensure consistent information about potential risks and benefits is available to women in multiple formats. These will be available from the feasibility study website, https://optibreech.uk.

Theoretical training will also be provided on-line through a Learning Management System platform, so that completion and comprehension testing can be tracked. Face-to-face training will focus on hands-on manoeuvres and occur for limited amounts of time and limited participants, with protective precautions in place. Cascade training is already an integral part of the intervention, as it enables those responsible for delivering the intervention to consolidate their own skills,^68^ so the research team will have face-to-face contact with as few local team members as needed.

## Peer and Ethical Review

The study design was peer reviewed by an external expert panel and the NIHR selection panel as part of the process of gaining NIHR grant funding. Peer review and PPI work concerning specific aspects of the study has continued during the protocol-writing stage. The protocol was reviewed and approved by all members of the research team. The entire protocol was externally reviewed by an ethics specialist within the NIHR Research Design Service London, with a favourable opinion, and the Principal Investigators from sites most likely to qualify for randomisation readiness.

## Distress and concerns

While to some professionals, enabling vaginal breech births to occur will feel like a change in practice, this protocol is completely compatible with the current RCOG^16^ and NICE^72^ guidelines concerning the management of breech presentation at term, both of which promote established principles of informed consent. However, as outlined in the Introduction, current habits of practice often do not follow these guidelines, and this has led to tension and discontent among women who wish to have this choice, and between professionals.

For women, is a risk that participation in this research may expose participants to the knowledge that national guidelines are not being followed. This may result in mistrust between women and clinicians. Additionally, participants may become aware of potential risks of current practice which they may be unaware of. In order to address this potential, all OptiBreech care members receive training about counselling participants in a way that is as neutral as possible and minimises this tension. We also make this training available to all staff within participating sites, regardless of their role on the team. Our Participant Information Sheets are also neutral.

For service users involved in either arm of our research, participation may make them feel guilty if they have previously experienced tragic or worrying births. Their participation in this research may lead them to feel they perhaps could have avoided them by asking for, insisting on or recognising a better method of delivery. We have had very open and honest discussions with lay members of our research team about how to approach this risk. For example, one woman on our team has lost her baby following a planned vaginal breech birth, and another has experienced anaphylactic shock and significant postnatal complications following a caesarean section for breech presentation. While aware that this could change at any time, both currently feel that contributing to this research helps them feel they are making a difference for future women, which outweighs their negative memories. If such distress is disclosed, we will immediately refer the participant to a local Birth Reflections service, which is designed for such events. Further need for counselling will be identified locally and arranged if necessary.

Additionally, there is potential for staff to feel the research team is judging and/or criticising their expertise or professionalism and the way they currently practice. Staff may also feel guilty if they have previously been involved with an adverse outcome that they now feel could have been prevented and may also need support. Care will be taken to reassure staff that the intention is to test an innovation that may potentially improve outcomes, rather than to take issue with care that has been delivered according to current standards. We will also seek to establish a ‘learning culture’ by encouraging reflection on every birth, including what could have been done better, so that staff feel safe to explore these issues without fearing they will be considered incompetent. Feedback from PIs and staff members in OptiBreech 1 so far indicates that the staff experience of participating in the research so far has been very positive. We have had positive feedback particularly from our reflective webinars, covering learning points arising in sites participating in OptiBreech 1. Our formal qualitative implementation evaluation is on-going.

## PPI & Ethics

The same potential exists within PPI work that recounting past experiences of difficult births may cause distress, and indeed this has happened throughout the project to date, so we have had to be very careful about our follow-up support arrangements. PPI members may disclose poor/negligent care that puts present women at risk of harm. Giving birth is a very personal yet very common experience, and similar sensitivities may also be raised within the research team. Therefore, PPI contributors, Trial Steering Committee members and Co-researchers are given clear guidance about their role and the importance of maintaining confidentiality. Information about how to alert the CI or RA that they require support is also provided, and the same procedures will be followed as for participants should either distress or concerns arise.

In response to PPI participants expressing the isolation they felt while planning their own breech births and a wish for more opportunities for peer support, an OptiBreech FaceBook group has been created, which is open only to participants and professionals involved in the project. The group is facilitated by a PPI lead who has previously completed training on listening skills and receives guidance from the research team. All women are given information about this opportunity at enrolment in the study.

The most significant issue identified through PPI work concerning the design of this research was around informed consent for randomisation. Service users were concerned about women being informed about a specialist team being available but being unable to access this if randomised to the control arm. In response, the design has been modified to a Trial Within Cohort (TWiC) model,^57^ which uses a patient-centred two-stage consent process, in which participants are only provided with information that is relevant to them.^73^ Feedback suggests this will help resolve this tension while preserving the scientific integrity of the investigation. The PPI group has reviewed the specific consent form we are proposing to use in this research, which was revised with their feedback and received a favourable opinion from those who reviewed it.

## Accessibility

In this feasibility study, we have not budgeted for translated materials. This is partially because the informational materials may change due to the feasibility work prior to a substantive trial. Our use of video Participant Information helps to a small degree because many women understand spoken English but do not necessarily understand written English. However, we will also ensure that each participating site has mechanisms in place to use translation services to enable women to participate regardless of their English literacy. Additionally, we will provide audio versions of the Participant Information Sheet and Consent Form, and it will be considered acceptable for a member of the local research team to assist women to complete the online form by enabling them to respond verbally. Finally, we aim to identify the leading languages other than English used in the participating sites in order to plan and budget for translations should a substantive trial be feasible.

# Study Oversight Arrangements

The study is sponsored by King’s College London.

## Trial Management Group (TMG)

The Trial Management Group will monitor the day-to-day running of the feasibility and will meet on a regular basis either in person or via Microsoft Teams.

Members of the TMG will include:

Dr Shawn Walker (CI)

Tisha Dasgupta (RA)

Prof Andrew Shennan (Co-I)

Sarah Hunter (Service User, Co-I)

Prof Jane Sandall (Co-I)

Prof Julia Fox-Rushby (Co-I, Health Economics)

Dr Kirsty Logan (Co-I, Statistical support)

## Trial Steering Committee (TSC)

The role of the TSC is to provide the overall supervision of the study. All SAEs and details will be reported to the TSC and Sponsor and REC when relevant as directed by the TSC. The TSC will monitor the progress of the study and conduct and advise on its scientific credibility. In this early stage of feasibility testing, the TSC will also fulfil the role of a Data Monitoring Committee and include an independent statistician. The TSC ultimately carries the responsibility for deciding whether a substantial trial is feasible.

A TSC charter will be agreed at the first TSC meeting to document how the committee will operate.

Members of the TSC are as follows:

Prof Soo Down (Chair)

Mr Kim Hinshaw

Statistician: TBA

Phoebe Roberts, London, Service User

[observer from the NIHR]

## Meeting schedule

One meeting will occur in the first three months of study opening, to review accumulated data from OptiBreech 1, prior to the start of randomisation in this study. Another meeting will occur after the close of recruitment. Following the meeting, a report and recommendation regarding the feasibility of conducting a full RCT will be submitted to the Sponsor.

# Financing and insurance

## Financing

The study is funded by the National Institute for Health Research (NIHR).

Each site (with the exception of the lead site) participating in the study will receive £150 per ***randomised*** recruit, up to a maximum of £3000 (20 recruits). This funding is intended to compensate the Trust for the time spent training their OptiBreech team and any on-call payments they have needed to put in place.

## Insurance

The Sponsor are responsible for ensuring proper provision has been made for insurance or indemnity to cover their liability of the Chief Investigator and staff.

The following arrangements are in place to fulfil the Sponsor’ responsibilities:

- The protocol has been designed by the Chief Investigator and researchers employed by the University and collaborators. The University has insurance in place (which includes no-fault compensation) for negligent harm caused by poor protocol designed by the Chief Investigator and researchers employed by the University.
- Sites participating in the study will be liable for clinical negligence and other negligent harm to individuals taking part in the study and covered by the duty of care owed to them by the Sites concerned. The Sponsor require individual sites participating in the study to arrange for their own insurance or indemnity in respect of these liabilities.
- Sites which are part of the United Kingdom’s Nation Health Service will have the benefits of NHS Indemnity.

# Reporting and Dissemination

## Transparency and openness strategy

We have adopted a multi-dimensional dissemination strategy consistent with the HRA’s standards, “Make it Public: Transparency and openness in health and social care research.”^74^ A dissemination strategy will be operational throughout this project, drawing on PPI, professional and policy networks and will involve the Trial Steering Committee (TSC) expert panel made up of service users, midwives, managers, academic collaborators and commissioners, as per the HRA guidance. As each aspect of the study is completed, the PPI group and TSC will be informed and the strategy discussed. Popular social media outputs will be utilised to share knowledge and advertise published findings.

In addition, the study website, https://optibreech.uk, will be used to disseminate updates about study progress and outcomes, including links to published papers and a brief, accessible summary of the findings. During the consent process, participants will be informed about how they will hear of the results of the study, e.g. through the OptiBreech website, via the FaceBook involvement group, or through other means.

## Authorship Policy

Ownership of the data arising from this is set out in the Organisation Information Document and an authorship policy will be developed. On completion of the study, the study data will be analysed and tabulated, and a clinical study report will be prepared in accordance with GCP guidelines.

## Publications

The study report will be used for publication and presentation at scientific meetings. The results of the study and any protocol deviations will be published in writing by the team headed by the Chief Investigator, which will report to the Trial Management Committee. Individual investigators may be able to produce oral reports with the permission of the Trial Management Committee.

Summaries of results will also be made available to Investigators for dissemination within their Trusts.

The entire project will be written up for publication. Findings for each section will be prepared for conference presentations and publication in peer reviewed journals such as 'The Lancet, 'PLoS ONE', 'Trials, and 'BMC Pregnancy and Childbirth'. A study report and summary will be prepared and submitted to the NIHR.

Anonymised raw data will be made available as supplementary material in open-access publications, in accordance with the NIHR publication guidance, and stored in a repository for longer-term availability, in accordance with WHO and ICMJE guidance.

# References

1. Angood PB, Armstrong EM, Ashton D, et al. Blueprint for action: steps toward a high-quality, high-value maternity care system. *Womens Health Issues*. 2010;20(1 Suppl):S18-49. doi:10.1016/j.whi.2009.11.007

2. Guittier M-J, Bonnet J, Jarabo G, Boulvain M, Irion O, Hudelson P. Breech presentation and choice of mode of childbirth: a qualitative study of women’s experiences. *Midwifery*. 2011;27(6):e208-13. doi:10.1016/j.midw.2010.08.008

3. Homer, Watts, Petrovska, Sjostedt, Bisits. Women’s experiences of planning a vaginal breech birth in Australia. *BMC Pregnancy Childbirth*. 2015;15(1):89. doi:10.1186/s12884-015-0521-4

4. Petrovska K, Watts NP, Catling C, Bisits A, Homer CSE. Supporting Women Planning a Vaginal Breech Birth: An International Survey. *Birth*. 2016;43(4):353-357. doi:10.1111/birt.12249

5. Sloman R, Wanat M, Burns E, Smith L. Midwives’ views, experiences and feelings of confidence surrounding vaginal breech birth: A qualitative study. *Midwifery*. 2016;41:61-67. doi:10.1016/j.midw.2016.07.015

6. van Roosmalen J, Meguid T. The dilemma of vaginal breech delivery worldwide. *Lancet*. 2014;383(9932):1863-1864. doi:10.1016/S0140-6736(14)60618-8

7. Petrovska K, Watts NP, Catling C, Bisits A, Homer CS. ‘Stress, anger, fear and injustice’: An international qualitative survey of women’s experiences planning a vaginal breech birth. *Midwifery*. 2017;44(0):41-47. doi:10.1016/j.midw.2016.11.005

8. Lightfoot K. Women’s experiences of undiagnosed breech birth and the effects on future childbirth decisions and expectations. Published online 2018. http://eprints.uwe.ac.uk/33278

9. Menakaya UA, Trivedi A. Qualitative assessment of women’s experiences with ECV. *Women and Birth*. 2013;26(1):e41-e44. doi:10.1016/j.wombi.2012.09.001

10. Say R, Thomson R, Robson S, Exley C. A qualitative interview study exploring pregnant women’s and health professionals’ attitudes to external cephalic version. *BMC Pregnancy Childbirth*. 2013;13(1):4. doi:10.1186/1471-2393-13-4

11. Watts NP, Petrovska K, Bisits A, Catling C. This baby is not for turning: Women’s experiences of attempted external cephalic version. *BMC Pregnancy Childbirth*. 2016;16(1):248. doi:10.1186/s12884-016-1038-1

12. Impey L, Murphy D, Griffiths M, Penna L, on behalf of the Royal College of Obstetricians and Gynaecologists. External Cephalic Version and Reducing the Incidence of Term Breech Presentation. *BJOG An Int J Obstet Gynaecol*. 2017;124(7):e178-e192. doi:10.1111/1471-0528.14466

13. Bogner G, Hammer BE, Schausberger C, Fischer T, Reisenberger K, Jacobs V. Patient satisfaction with childbirth after external cephalic version. *Arch Gynecol Obstet*. 2014;289(3):523-531. doi:10.1007/s00404-013-3007-5

14. Yeoh SGJ, Rolnik DL, Regan JA, Lee PYA. Experience and confidence in vaginal breech and twin deliveries among obstetric trainees and new specialists in Australia and New Zealand. *Aust New Zeal J Obstet Gynaecol*. Published online December 18, 2018. doi:10.1111/ajo.12931

15. Hofmeyr GJ, Hannah M, Lawrie TA. Planned caesarean section for term breech delivery. In: Hofmeyr GJ, ed. *Cochrane Database of Systematic Reviews*. John Wiley & Sons, Ltd; 2015:Art. No.: CD000166. doi:10.1002/14651858.CD000166.pub2

16. Impey L, Murphy D, Griffiths M, Penna L, on behalf of the Royal College of Obstetricians and Gynaecologists. Management of Breech Presentation. *BJOG An Int J Obstet Gynaecol*. 2017;124(7):e151-e177. doi:10.1111/1471-0528.14465

17. Macharey G, Ulander VM, Kostev K, Väisänen-Tommiska M, Ziller V. Emergency peripartum hysterectomy and risk factors by mode of delivery and obstetric history: A 10-year review from Helsinki University Central Hospital. *J Perinat Med*. 2015;43(6):721-728. doi:10.1515/jpm-2013-0348

18. Kok M, Gravendeel L, Opmeer BC, van der Post JAM, Mol BWJ. Expectant parents’ preferences for mode of delivery and trade-offs of outcomes for breech presentation. *Patient Educ Couns*. 2008;72(2):305-310. doi:10.1016/j.pec.2008.04.008

19. Abdessalami S, Rota H, Pereira GD, Roest J, Rosman AN. The influence of counseling on the mode of breech birth: A single-center observational prospective study in The Netherlands. *Midwifery*. 2017;55:96-102. doi:10.1016/j.midw.2017.09.012

20. Mohajer M. Results of a telephone survey. Management of the Term Breech.

21. Hickland P, Gargan P, Simpson J, McCabe N, Costa J. A novel and dedicated multidisciplinary service to manage breech presentation at term; 3 years of experience in a tertiary care maternity unit. *J Matern Neonatal Med*. 2018;31(22):3002-3008. doi:10.1080/14767058.2017.1362382

22. Kidd L, Rivers A, George R, Singh N, Yentis S. PA.12 Development of a dedicated breech service in a London teaching hospital. *Arch Dis Child - Fetal Neonatal Ed*. 2014;99(Suppl 1):A20.3-A21. doi:10.1136/archdischild-2014-306576.57

23. Louwen F, Daviss B, Johnson KC, Reitter A. Does breech delivery in an upright position instead of on the back improve outcomes and avoid cesareans? *Int J Gynecol Obstet*. 2017;136(2):151-161. doi:10.1002/ijgo.12033

24. Reitter A, Doehring N, Maden Z, Hessler PA, Misselwitz B. Is it Reasonable to Establish an Independently Managed Obstetric Unit in a Small Hospital and Does it Result in Measurable Changes in Quality of Maternity Care? *Z Geburtshilfe Neonatol*. 2019;223(3):147-156. doi:10.1055/a-0749-9024

25. NHS Digital. NHS Maternity Statistics, 2018-2019. Published 2019. Accessed January 17, 2020. https://digital.nhs.uk/data-and-information/publications/statistical/nhs-maternity-statistics/2018-19

26. Lafitte A-S, Dolley P, Le Coutour X, et al. Rate of caesarean sections according to the Robson classification: Analysis in a French perinatal network – Interest and limitations of the French medico-administrative data (PMSI). *J Gynecol Obstet Hum Reprod*. 2018;47(2):39-44. doi:10.1016/J.JOGOH.2017.11.012

27. NICE. Caesarean birth. NICE Guideline 192. Published 2021. https://www.nice.org.uk/guidance/ng192

28. MBRRACE. *Saving Lives, Improving Mothers’ Care: Lessons Learned to Inform Maternity Care from the UK and Ireland Confidential Enquiries into Maternal Deaths and Morbidity 2014-2016*.; 2018. https://www.npeu.ox.ac.uk/downloads/files/mbrrace-uk/reports/MBRRACE-UK Maternal Report 2018 - Web Version.pdf

29. Vlemmix F, Kazemier B, Rosman A, et al. 764: Effect of increased caesarean section rate due to term breech presentation on maternal and fetal outcome in subsequent pregnancies. *Am J Obstet Gynecol*. 2013;208(1):S321. doi:10.1016/j.ajog.2012.10.102

30. Macharey G, Toijonen A, Hinnenberg P, Gissler M, Heinonen S, Ziller V. Term cesarean breech delivery in the first pregnancy is associated with an increased risk for maternal and neonatal morbidity in the subsequent delivery: a national cohort study. *Arch Gynecol Obstet*. 2020;302(1):85-91. doi:10.1007/s00404-020-05575-6

31. Hemelaar J, Lim LN, Impey LW. The Impact of an ECV Service is Limited by Antenatal Breech Detection: A Retrospective Cohort Study. *Birth*. 2015;42(2):165-172. doi:10.1111/birt.12162

32. Pergialiotis V, Vlachos DG, Rodolakis A, Haidopoulos D, Thomakos N, Vlachos GD. First versus second stage C/S maternal and neonatal morbidity: a systematic review and meta-analysis. *Eur J Obstet Gynecol Reprod Biol*. 2014;175(4):15-24. doi:http://dx.doi.org/10.1016/j.ejogrb.2013.12.033

33. Watson HA, Carter J, David AL, Seed PT, Shennan AH. Full dilation cesarean section: a risk factor for recurrent second-trimester loss and preterm birth. *Acta Obstet Gynecol Scand*. 2017;96(9):1100-1105. doi:10.1111/aogs.13160

34. Kotaska A. Commentary: routine cesarean section for breech: the unmeasured cost. *Birth*. 2011;38(2):162-164. doi:10.1111/j.1523-536X.2011.00468.x

35. Magro M. Five years of cerebral palsy claims: A thematic review of NHS Resolution data. Published 2017. Accessed August 24, 2019. https://resolution.nhs.uk/resources/five-years-of-cerebral-palsy-claims/

36. NICE. *Caesarean Section. NICE Clinical Guideline.* National Collaborating Centre for Women’s and Children’s Health; 2011.

37. Hofmeyr GJ, Kulier R, West HM. External cephalic version for breech presentation at term. *Cochrane database Syst Rev*. 2015;(4):CD000083. doi:10.1002/14651858.CD000083.pub3

38. Walker S, Scamell M, Parker P. Principles of physiological breech birth practice: A Delphi study. *Midwifery*. 2016;43(0):1-6. doi:10.1016/j.midw.2016.09.003

39. Su M, McLeod L, Ross S, et al. Factors associated with adverse perinatal outcome in the Term Breech Trial. *Am J Obstet Gynecol*. 2003;189(3):740-745. doi:10.1067/S0002-9378(03)00822-6

40. Melo P, Georgiou EX, Hedditch A, Ellaway P, Impey L. External cephalic version at term: a cohort study of 18 years’ experience. *BJOG An Int J Obstet Gynaecol*. 2019;126(4):493-499. doi:10.1111/1471-0528.15475

41. Vlemmix F, Bergenhenegouwen L, Schaaf JM, et al. Term breech deliveries in the Netherlands: did the increased cesarean rate affect neonatal outcome? A population-based cohort study. *Acta Obstet Gynecol Scand*. 2014;93(9):888-896. doi:10.1111/aogs.12449

42. Rosman AN, Vlemmix F, Ensing S, et al. Mode of childbirth and neonatal outcome after external cephalic version: A prospective cohort study. *Midwifery*. 2016;39:44-48. doi:10.1016/j.midw.2016.02.014

43. Wastlund D, Moraitis AA, Dacey A, Sovio U, Wilson ECF, Smith GCS. Screening for breech presentation using universal late-pregnancy ultrasonography: A prospective cohort study and cost effectiveness analysis. Myers JE, ed. *PLoS Med*. 2019;16(4):e1002778. doi:10.1371/journal.pmed.1002778

44. Jensen VM, Wüst M. Can Caesarean section improve child and maternal health? The case of breech babies. *J Health Econ*. 2014;39:289-302. doi:10.1016/j.jhealeco.2014.07.004

45. Hannah ME, Hannah WJ, Hewson SA, Hodnett ED, Saigal S, Willan AR. Planned caesarean section versus planned vaginal birth for breech presentation at term: a randomised multicentre trial. Term Breech Trial Collaborative Group. *Lancet*. 2000;356(9239):1375-1383.

46. Bogner G, Strobl M, Schausberger C, Fischer T, Reisenberger K, Jacobs VR. Breech delivery in the all fours position: a prospective observational comparative study with classic assistance. *J Perinat Med*. 2015;43(6):707-713. doi:10.1515/jpm-2014-0048

47. Walker S, Scamell M, Parker P. Standards for maternity care professionals attending planned upright breech births: A Delphi study. *Midwifery*. 2016;34:7-14. doi:10.1016/j.midw.2016.01.007

48. Walker S, Reading C, Siverwood-Cope O, Cochrane V. Physiological breech birth: Evaluation of a training programme for birth professionals. *Pract Midwife*. 2017;20(2):25-28.

49. Walker S, Parker P, Scamell M. Expertise in physiological breech birth: A mixed-methods study. *Birth*. 2018;45(2):202-209. doi:10.1111/birt.12326

50. Moore GF, Audrey S, Barker M, et al. Process evaluation of complex interventions: Medical Research Council guidance. *BMJ*. 2015;350:h1258. doi:10.1136/bmj.h1258

51. Spillane E, Walker S. Case study supporting continuity of care models for breech presentation at or near term. *Pract Midwife*. Published online 2019:36-37.

52. Derisbourg S, Costa E, De Luca L, et al. Impact of implementation of a breech clinic in a tertiary hospital. *BMC Pregnancy Childbirth*. 2020;20(1):435. doi:10.1186/s12884-020-03122-4

53. Mattiolo S, Spillane E, Walker S. Physiological breech birth training: An evaluation of clinical practice changes after a one‐day training program. *Birth*. Published online June 23, 2021:birt.12562. doi:10.1111/birt.12562

54. Walker S, Dasgupta T, Halliday A, Reitter A. Development of a core outcome set for effectiveness studies of breech birth at term (Breech-COS): A systematic review on variations in outcome reporting. *Eur J Obstet Gynecol Reprod Biol*. 2021;263:117-126. doi:10.1016/j.ejogrb.2021.06.021

55. Walker S, Dasgupta T, Halliday A, Reitter A. Development of a core outcome set for effectiveness studies of breech birth at term (Breech-COS): a systematic review on variations in outcome reporting. *Rev*. Published online 2021.

56. Ford I, Norrie J. Pragmatic Trials. Drazen JM, Harrington DP, McMurray JJV, Ware JH, Woodcock J, eds. *N Engl J Med*. 2016;375(5):454-463. doi:10.1056/NEJMra1510059

57. Bibby AC, Torgerson DJ, Leach S, Lewis-White H, Maskell NA. Commentary: Considerations for using the “Trials within Cohorts” design in a clinical trial of an investigational medicinal product. *Trials*. 2018;19(1):18. doi:10.1186/s13063-017-2432-3

58. Hoffmann TC, Glasziou PP, Boutron I, et al. Better reporting of interventions: template for intervention description and replication (TIDieR) checklist and guide. *BMJ*. 2014;348:g1687. doi:10.1136/bmj.g1687

59. Dencker A, Taft C, Bergqvist L, Lilja H, Berg M. Childbirth experience questionnaire (CEQ): Development and evaluation of a multidimensional instrument. *BMC Pregnancy Childbirth*. 2010;10(1):1-8. doi:10.1186/1471-2393-10-81

60. Walker KF, Wilson P, Bugg GJ, Dencker A, Thornton JG. Childbirth experience questionnaire: Validating its use in the United Kingdom. *BMC Pregnancy Childbirth*. 2015;15(1):1-8. doi:10.1186/s12884-015-0513-4

61. Evans J, Smith A, Gibbons C, Alonso J, Valderas J. The National Institutes of Health Patient-Reported Outcomes Measurement Information System (PROMIS): a view from the UK. *Patient Relat Outcome Meas*. 2018;Volume 9:345-352. doi:10.2147/prom.s141378

62. Squires J, Bricker D. *Ages & Stages Questionnaires®, Third Edition (ASQ®-3): A Parent-Completed Child Monitoring System*. Paul H. Brookes Publishing Co., Inc.; 2009.

63. Lewis R, Todd R, Newton M, et al. The implementation and utility of patient screening logs in a multicentre randomised controlled oncology trial. *Trials*. 2020;21(1):629. doi:10.1186/s13063-020-04559-w

64. Walker S. Undiagnosed breech: Towards a woman-centred approach. *Br J Midwifery*. 2013;21(5):316-322. Accessed March 7, 2014. http://www.intermid.co.uk/cgi-bin/go.pl/library/article.cgi?uid=98340;article=BJM_21_5_316_322

65. Walker S, Cochrane V. Unexpected breech: what can midwives do? *Pract Midwife*. 2015;18(10):26-29.

66. *Obtaining Valid Consent to Participate in Perinatal Research Where Consent Is Time Critical*.; 2016.

67. EPOC Taxonomy. Effective Practice and Organisation of Care (EPOC). Published 2015. Accessed June 9, 2020. https://epoc.cochrane.org/sites/epoc.cochrane.org/files/public/uploads/taxonomy/epoc_taxonomy.pdf

68. Walker S, Scamell M, Parker P. Deliberate acquisition of competence in physiological breech birth: A grounded theory study. *Women and Birth*. 2018;31(3):e170-e177. doi:10.1016/j.wombi.2017.09.008

69. Walker S. No more ‘ hands off the breech .’ *Pract Midwife*. 2020;6(June):1-8. Accessed July 25, 2020. https://www.all4maternity.com/no-more-hands-off-the-breech/

70. Shields N, Turnbull D, Reid M, Holmes A, McGinley M, Smith LN. Satisfaction with midwife-managed care in different time periods: A randomised controlled trial of 1299 women. *Midwifery*. 1998;14(2):85-93. doi:10.1016/S0266-6138(98)90003-1

71. Goffinet F, Carayol M, Foidart J-M, et al. Is planned vaginal delivery for breech presentation at term still an option? Results of an observational prospective survey in France and Belgium. *Am J Obstet Gynecol*. 2006;194(4):1002-1011. doi:10.1016/j.ajog.2005.10.817

72. NICE. Intrapartum care for women with existing medical conditions or obstetric complications and their babies(NG121). Published online 2019.

73. Relton C, Torgerson D, O’Cathain A, Nicholl J. Rethinking pragmatic randomised controlled trials: introducing the &quot;cohort multiple randomised controlled trial&quot; design. *BMJ*. 2010;340:c1066. doi:10.1136/bmj.c1066

74. Health Research Authority. Make it Public: transparency and openness in health and social care research. Published 2020. Accessed September 23, 2020. https://www.hra.nhs.uk/planning-and-improving-research/policies-standards-legislation/research-transparency/make-it-public-transparency-and-openness-health-and-social-care-research/

75. MEASURE Evaluation. Family Planning and Reproductive Health Indicators Database. Accessed April 2, 2020. https://www.measureevaluation.org/prh/rh_indicators

# Definitions

Breech – ‘Breech’ refers to a breech-presenting fetus, that is lying in a longitudinal position, with buttocks, feet or knees closest to the cervical os.

CI – Chief Investigator. On behalf of the Sponsor, the CI has overall responsibility for the design and conduct of the study. The CI also has co-ordinating responsibility for reporting adverse events to the Sponsor and to the relevant Research Ethics Committee (REC).

CRF – Care Report Form. A paper or electronic questionnaire used to collect date on/from each participant.

CS – Caesarean section

R&D – Research and Development Office

GCP – Good Clinical Practice. Every clinician taking consent to participate in this research should have completed Good Clinical Practice training.

Independent Data Monitoring Committee (IDMC) – An independent data-monitoring committee that may be established by the sponsor to assess at intervals the progress of a clinical trial, the safety data, and the critical efficacy endpoints, and to recommend to the sponsor whether to continue, modify, or stop a trial.

OptiBreech Team – The care in this study is delivered by an ‘OptiBreech Team’, in which each member has undertaken physiological breech birth training and meets the OptiBreech Proficiency Criteria (p36).

PBB – Physiological breech birth. A physiological breech birth is a vaginal breech birth in which the woman is encouraged to remain upright and active throughout her labour and able to assume the position of her choice for the birth, including upright postures. Guidance for those attending PBBs is based on research on ‘physiological breech birth’ and includes recommended time limits around late second stage, and recommended interventions if these are exceeded. VBBs within the OptiBreech care pathway are managed according to PBB principles.

Perinatal death – A fetal death (stillbirth) or an early neonatal death (0-6 days). (Evaluation outcome definitions are taken from the MEASURE Evaluation Family Planning and Reproductive Health Indicators Database.^75^)

PI – Principle Investigator. Each site will have 1 PI, who should be either the Breech Lead Obstetrician or the Breech Lead Midwife. They will be responsible for overseeing the local data collection and for informing the CI of all SAEs that occur at the site. Along with the other Lead (Obstetrician or Midwife), they will also be responsible for clinically leading the delivery of the intervention.

PMR – Perinatal mortality rate, defined as the number of perinatal deaths per 1000 total births.

R&D – Research and Development

RA – Research Assistant

REC – Research Ethics Committee

SOP – Standard Operation Procedure

Term – ‘Term’ refers to a term pregnancy, defined as a gestation greater than 36 weeks 6 days and less than 42 weeks 0 days.

# Appendix 1: OptiBreech Practice Guideline

## Background

Women who choose to plan a vaginal breech birth want that birth to be as safe as possible for both their baby and themselves. They have been fully counselled about the potential need for assistance or an intrapartum CS, and these should be used as necessary when the safety of the clinical situation is uncertain.

OptiBreech care is based on evidence that care from a proficient practitioner, throughout the care pathway, is likely to improve neonatal outcomes and increase the vaginal birth rate among women who desire to give birth vaginally. Where OptiBreech-specific principles of care are not covered in this guideline, clinicians should use national and local guidelines to guide practice.

## Definition of proficiency

A professional is considered currently proficient to lead OptiBreech care if they have:

1. Participated in 6 hours of evaluated physiological breech birth training;
2. Attended at least 10 vaginal breech births, including resolution of complications using manual manoeuvres;
3. Attended or taught in simulation at least 3 vaginal breech births within the past year;
4. Delivered physiological breech birth training at least once within the past year, including reflective reviews of births attended;
5. Completed an OptiBreech Proficiency self-assessment and indicated that they feel competent to implement the OptiBreech Practice Guideline at vaginal breech births where they are the designated clinical lead, and this has been confirmed by the Breech Leads.

A fully proficient OptiBreech team practitioner should be present for a minimum throughout second stage and have overall clinical responsibility for each birth within the OptiBreech care pathway. The role of the OptiBreech team member is to provide clinical leadership as part of a team. They will not normally also be responsible for providing hands-on care, unless another OptiBreech team member is also present.

## Commence breech pathway

**36 weeks gestation**

The OptiBreech care pathway begins at 36 weeks of pregnancy. This is because breech presentation at term, in the absence of other complications, is regarded as a ‘variation of normal.’ Women are **not** encouraged to try to turn their babies, through moxibustion, postural exercises, acupuncture or external cephalic version. However, if they are drawn to those modalities, they are not discouraged from using them and should be given safety advice and support.

When women are booked for a presentation scan, they should be offered information about the OptiBreech Care study prior to their scan appointment. This is to enable them to make an informed decision about participation in the study.

In the OptiBreech care study, if women are referred for breech care prior to 36 weeks, they consent to participation in the study following diagnosis and are randomised to OptiBreech care, they should be counselled following randomisation as usual. Counselling should follow the Pro Forma included in the CRF.

They should be offered the following 3 options:

1. Carrying on as normal and continuing to plan a vaginal birth, while receiving the remainder of their pregnancy and birth care from the OptiBreech team, co-ordinated by the Breech Specialist Midwife;
2. Attempting an ECV according to the local guideline but remaining under OptiBreech care and planning a VBB if it is unsuccessful; or
3. Declining OptiBreech care and being referred back to the ‘usual care’ pathway, including the usual care ECV service or planned CS and pregnancy care by their named midwife.

Women are able to return to the OptiBreech pathway at any time if they change their mind and wish to plan a VBB. Women within the OptiBreech pathway are also able to return to ‘usual care’ or plan a CS at any time if they change their mind.

## Biometric Growth Ultrasound Scans

Women whose babies are diagnosed in breech presentation at the end of pregnancy should be offered a full biometric growth ultrasound scan, performed by a sonographer or other professional with equivalent qualifications. Ideally, this should be performed around 36 weeks of pregnancy. Decisions about mode of birth and the timing of an end-of-pregnancy elective caesarean section or induction, in the event of no labour, should be made on the basis of this initial ultrasound and expected growth trajectory. Additional growth scans should only be performed where standard antenatal screening suggests concerns about fetal growth or well-being, as the accuracy of such scans diminish in later gestations. Point-of-care bedside scans should be used to inform care as needed.

Women who decline a full biometric growth scan are still eligible to participate in the OptiBreech Care study, including randomisation, if otherwise eligible based on other clinical findings.

## Labour Care

All members of the intrapartum team should be made aware of this guideline and ideally should have received information about it during their mandatory training. Hands-on labour care should be provided by someone who has received physiological breech birth training, either through the OptiBreech training or as part of their mandatory training package. One member of the intrapartum care team who has completed the enhanced OptiBreech training should be designated the role of lead, and it is their responsibility to maintain the ‘helicopter view’ of the birth.

### Monitoring

Follow the NICE Guideline on Intrapartum Care for Healthy Women and Babies for monitoring and assessment of progress in labour. Continuous fetal monitoring should be offered, but a woman’s preference for intermittent monitoring should be respected. Where external monitoring is expected to be difficult (e.g. elevated BMI, longer second stage, etc.), consider use of a fetal electrode, taking care to avoid the genital area on application.

### Progress in second stage of labour

Descent is assessed by the station of the fetal buttocks. Provided fetal heart monitoring shows no evidence of compromise nor diminished reserves, a passive second stage of up to 2 hours is acceptable and advised if the woman has an epidural in situ. After 2 hours of passive second stage, the buttocks should be visible at the introitus; otherwise, a CS should be recommended. Following descent to ‘rumping’ (+3 station, anus and both buttocks visible), the birth should normally be complete within 7 minutes.

A passive second stage is also acceptable with no epidural, but do not instruct women to resist a spontaneous urge to push. If after one hour of active pushing the buttocks are not visible at the introitus consider the need for a CS unless the fetal heart rate is completely normal and there has been considerable descent in this time. Risk of an adverse outcome increases with each 30 minutes of active pushing; this should be considered in light of evidence of fetal well-being.

During emergence, maintain awareness of normal intervals. Accurate fetal heart rate monitoring is very difficult, and cord occlusion very likely. Use the Physiological Breech Birth Algorithm as a guide for which interventions are indicated and when. Most vaginal breech births are complete within 7 minutes of ‘rumping’ (both buttocks remaining visible on the perineum between contractions, or +3 station), including time for hands-on interventions if indicated. An episiotomy is not indicated until this point, if at all.

If an episiotomy does not result in the birth of the pelvis and clear progress, an urgent CS is indicated. Care should be taken to elevate the fetal pelvis using pressure on the pelvic bones only, to avoid perineal or genital damage. A fetal pillow may assist in preventing trauma, but elevation is not expected to be difficult unless the pelvis has been born.

If a pause of 30 seconds or more occurs once the pelvis is born, encourage the woman to move and/or push actively. Do not instruct the woman to wait for the next contraction to push at this stage. If maternal movement and effort do not result in immediate progress, assume this is due to obstruction and assist the birth as appropriate. Once intervention has been initiated, the attendant should continue to assist the birth until the baby is born.

Where progress has been rapid up to the umbilicus, the birth should be complete within 3 minutes of this point, including time for hands-on assistance if indicated. A member of the intrapartum team should be designated to be prepared to assist the lead professional, where required (e.g. buttock lift, assisting with elevation to higher station if head is extended at inlet, applying fetal pillow, etc.).

## Neonatal Care

Breech presenting babies often appear depressed at birth due to acute cord compression at the end of labour. NICE guidance should be followed regarding optimal cord management. The cord MUST NOT be clamped prior to 1 minute following birth, unless the cord has ruptured or the FH is confirmed by stethoscope to be <60 bmp and not improving. This is to avoid the risk of a reflex bradycardia, to which breech babies appear particularly vulnerable. The neonatal team should be encouraged to come to the bedside to make this assessment. If neonatal condition indicates that resuscitation is necessary, inflation breaths should be initiated with a bag and mask, with the umbilical cord remaining intact. In most cases, the release of cord occlusion and placental resuscitation will lead to immediate improvement in neonatal condition. If inflation breaths are unsuccessful or further resuscitation is required, transfer the baby to the neonatal team.

A member of the neonatal team should be called to attend all vaginal breech births. A member of the intrapartum care team should be designated during labour with the role of obtaining cord blood samples from the intact cord and initiating resuscitation on the bed or beside it, using a bag and mask or bedside unit, should these be required.

## Special Clinical Situations

### Care of non-extended breech presentation

Very little high-quality evidence exists to guide care of non-extended (non-frank) breech presentation in labour. For women under OptiBreech care, care should conform to the Principles of Physiological Breech Birth, which focuses on careful evaluation. In these cases, the cervix will dilate with pressure from the fetal buttocks, regardless of where the legs are. In a full term, symmetrically grown fetus, the bitrochanteric diameter is also expected to be 10 cm.

For any non-extended breech presentation (flexed, semi-flexed, kneeling, dropped foot), counsel the woman about the increased risk of cord prolapse and encourage her to alert someone if she feels anything in her vagina. Offer intravenous cannulation. The increased risk of cord prolapse for breech is not associated with an increase in adverse outcomes, as long as it is anticipated and action taken as necessary. Monitor the fetal heart rate closely, according to standard guidelines.

If a foot is felt below the buttocks in labour, this is not an automatic indication for a CS in labour, unless the lie is not longitudinal. Assess descent according to the buttocks as usual, performing only the minimum number of vaginal examinations required. It is common for a flexed breech baby to drop a leg down as the cervix dilates and more space becomes available underneath the sacrum. Descent will normally not begin until the buttocks have fully dilated the cervix. The mechanisms will be similar to any other breech birth, with the sacrum descending in a transverse position.

When one or both knees present, the sacrum often descends in a posterior position. This is also not an automatic indication for a CS in labour. Advise the woman about the increased risk of cord prolapse as per above. The presenting part(s) will rotate on the perineum, and you should expect the sacrum to emerge in a transverse position as per usual. There is no need to extract the legs if progress is normal and there are no concerns about fetal condition. If progress arrests on the perineum and fetal leg extraction appears to be needed, care should be taken to sweep down the leg across the body, with buttocks remaining in situ until after extraction, and not to pull on the foot.

### Fetal size

Women should be advised that the strongest evidence for an increased perinatal risk is for small babies (<2.5 kg, <10^th^ centile). These babies are more likely to have an underlying problem, and even where size is constitutionally small, may have fewer reserves.

Women should be advised that the evidence from centres where upright breech birth is practiced does not indicate increased neonatal risk or maternal birth injuries for larger breech babies (>3.8 kg). However, an intrapartum caesarean section is more likely.

Fetal size should be considered holistically, taking account of the overall consistency of growth trajectory as an indication of fetal well-being and ability to cope with labour.

### Prior Caesarean Section

Women should be advised that outcomes for breech births after a prior caesarean section are similar to those for nulliparous women.

## Assessment of Fidelity

Each of these recommendations will be assessed using the information provided about births in the OptiBreech eCRF.
