# Supplementary material for: OptiBreech collaborative care versus standard care for women with a breech-presenting fetus at term: A pilot parallel group randomised trial to evaluate the feasibility of a randomised trial nested within a cohort
Source: PLoS One. 2023 Nov 15;18(11):e0294139. doi: 10.1371/journal.pone.0294139 (PMC10650999; doi:10.1371/journal.pone.0294139)
Supplement: Attachment — Submitted filename: Editorial checks.docx [file pone.0294139.s004.docx]

PONE-D-23-09332, Editor Checks, 29/3/22

OptiBreech collaborative care versus standard care for women with a breech-presenting fetus at term: a pilot parallel group randomised trial to evaluate the feasibility of a randomised trial nested within a cohort
Dr Shawn Walker

Dear Dr. Walker,

We've checked your submission and before we can proceed, we need you to address the following issues:

1. Please complete all items on the Clinical Studies Checklist that are relevant for your submission, by following this link: <https://eur03.safelinks.protection.outlook.com/?url=http%3A%2F%2Fjournals.plos.org%2Fplosone%2Fs%2Ffile%3Fid%3Ddc11%2FPLOSOne_Clinical_Studies_Checklist.docx&data=05%7C01%7Cshawn.walker%40kcl.ac.uk%7C0343358b36194dc70a0608db3038d2e4%7C8370cf1416f34c16b83c724071654356%7C0%7C0%7C638156793161735966%7CUnknown%7CTWFpbGZsb3d8eyJWIjoiMC4wLjAwMDAiLCJQIjoiV2luMzIiLCJBTiI6Ik1haWwiLCJXVCI6Mn0%3D%7C3000%7C%7C%7C&sdata=tFN9S%2BjwRj225x64Y5dulAv1k%2BGpW2%2FxFjGZpLPO6qE%3D&reserved=0> (Contact us at [plosone@plos.org](mailto:plosone@plos.org) if you cannot access the document.) There may be overlap between the checklist items and other queries listed below; please address any duplicated queries both in your response email and on the checklist itself. Upload the completed Clinical Studies Checklist as file type “Other” when you re-submit your manuscript. This document is for internal journal use only and will not be published if your article is accepted. The requested information will help us to assess whether your submission complies with PLOS ONE’s policies and adheres to applicable reporting standards. Note that your manuscript may be rejected if you provide incomplete or inadequate responses to the checklist questions and that changing the ‘Section/Category’ of your
article does not affect this requirement.

Completed. CONSORT checklist has been used because the purpose was to report the pilot trial. Please advise if you require the STROBE checklist in addition to this.

2. It has come to our attention that the manuscript seems to be a resubmission of the manuscript PMEDICINE-D-23-00153, which was previously considered by the journal PLOS Medicine.

It is a requirement by the journal that any manuscript resubmitted after a previous round of review is accompanied by a rebuttal letter outlining the response to the original reviewers’ comments, so that the editors can establish whether the original concerns have been addressed. Can you therefore please provide us with a point-by-point response to the comments provided by the original reviewers, outlining the revisions carried out since the original submission.

This was declared on submission. There was no rebuttal because the editors concluded the paper did not meet the criteria for PLOS Medicine, and their were no peer reviews.

As with all manuscripts submitted to the journal, yours was considered in light of other papers that we receive, and our aims for the journal. We are looking for papers of wide general interest which would lead to a substantial advance in clinical management or public health policy, or which provide a substantial new insight into the pathogenesis of disease, with a clear path toward clinical application. Within these categories, we prioritize research on conditions that contribute to a substantial proportion of the global burden of disease.

Now that we have had a chance to evaluate and discuss the paper, I regret to say that we do not think that it is appropriate for the journal given our editorial criteria.

While we cannot therefore consider your manuscript further for publication in PLOS Medicine, we suggest transferring your manuscript to  PLOS ONE instead.

3. Please upload a copy of your trial study protocol as a supporting information file. By the study protocol, we mean the complete and detailed plan for the conduct and analysis of the trial that the ethics committee approved before the trial began. Please send this in the original language. If this is in a language other than English, please also provide a translation. Please detail any deviations from this study protocol in the Methods section of your manuscript. Your study protocol will be made available to the editors and reviewers, and will be published as supporting information with your manuscript if accepted for publication. (If you do not agree to this, we will not be able to publish your manuscript). If you have formally published a study protocol for your trial in a journal then you should cite this in your manuscript, but you still need to send us the original document.

Uploaded.

We've returned your manuscript to your account. Please resolve these issues and resubmit your manuscript within 21 days. If you need more time, please email the journal office at [plosone@plos.org](mailto:plosone@plos.org). We are happy to grant extensions of up to one month past this due date. If we do not hear from you within 21 days, we will withdraw your manuscript.

Please log on to PLOS Editorial Manager at <https://eur03.safelinks.protection.outlook.com/?url=https%3A%2F%2Fwww.editorialmanager.com%2Fpone%2F&data=05%7C01%7Cshawn.walker%40kcl.ac.uk%7C0343358b36194dc70a0608db3038d2e4%7C8370cf1416f34c16b83c724071654356%7C0%7C0%7C638156793161735966%7CUnknown%7CTWFpbGZsb3d8eyJWIjoiMC4wLjAwMDAiLCJQIjoiV2luMzIiLCJBTiI6Ik1haWwiLCJXVCI6Mn0%3D%7C3000%7C%7C%7C&sdata=vMyjFqGYJFdjucJaL2Tly6Rqu7l6iMxbTILoXIEu7Ds%3D&reserved=0> to access your manuscript. You will find your manuscript in the 'Submissions Sent Back to Author' link under the New Submissions menu. Be sure to remove your previous manuscript file if you are uploading a new file in response to these requests. After you've made the changes requested above, please be sure to view and approve the revised PDF after rebuilding the PDF to complete the resubmission process.

We are requesting these changes to comply with the PLOS ONE submission guidelines (<https://eur03.safelinks.protection.outlook.com/?url=https%3A%2F%2Fjournals.plos.org%2Fplosone%2Fs%2Fsubmission-guidelines&data=05%7C01%7Cshawn.walker%40kcl.ac.uk%7C0343358b36194dc70a0608db3038d2e4%7C8370cf1416f34c16b83c724071654356%7C0%7C0%7C638156793161735966%7CUnknown%7CTWFpbGZsb3d8eyJWIjoiMC4wLjAwMDAiLCJQIjoiV2luMzIiLCJBTiI6Ik1haWwiLCJXVCI6Mn0%3D%7C3000%7C%7C%7C&sdata=ZxYAqGIi3Ydk%2B7hNU8MdODZrJv5w%2FshcYR4x8uKTizc%3D&reserved=0>). Please note that we won't send your manuscript for review until you have resolved the above requests. 

Thank you for submitting your work to PLOS ONE and supporting our mission of Open Science.


Kind regards,

Mia Vanessa Recto
PLOS ONE
