# Supplementary material for: OptiBreech collaborative care versus standard care for women with a breech-presenting fetus at term: A pilot parallel group randomised trial to evaluate the feasibility of a randomised trial nested within a cohort
Source: PLoS One. 2023 Nov 15;18(11):e0294139. doi: 10.1371/journal.pone.0294139 (PMC10650999; doi:10.1371/journal.pone.0294139)
Supplement: Attachment — Submitted filename: Response to Reviewers.docx [file pone.0294139.s005.docx]

**Response to Reviewers**

1. Please ensure that your manuscript meets PLOS ONE's style requirements, including those for file naming. The PLOS ONE style templates can be found at 
<https://journals.plos.org/plosone/s/file?id=wjVg/PLOSOne_formatting_sample_main_body.pdf> and 
<https://journals.plos.org/plosone/s/file?id=ba62/PLOSOne_formatting_sample_title_authors_affiliations.pdf>

**Checked and formatted per template.**

2. Thank you for stating the following in the Competing Interests section: 
"I have read the journal's policy and the authors of this manuscript have the following competing interests: SW and ES are co-Directors of Breech Birth Network, Community Interest Company, a not-for-profit social enterprise that delivers VBB training and supports research. They and other members of the OptiBreech Collaborative have received teaching fees and expenses for providing breech training."

Please confirm that this does not alter your adherence to all PLOS ONE policies on sharing data and materials, by including the following statement: ""This does not alter our adherence to  PLOS ONE policies on sharing data and materials.” (as detailed online in our guide for authors <http://journals.plos.org/plosone/s/competing-interests>).  If there are restrictions on sharing of data and/or materials, please state these. Please note that we cannot proceed with consideration of your article until this information has been declared.

Please include your updated Competing Interests statement in your cover letter; we will change the online submission form on your behalf.

**We have added this statement in our cover letter. There are no restrictions on data sharing.**

3. We note that you have referenced (Walker S, Spillane E, Stringer K, Meadowcroft A, Dasgupta T, Davies S, et al. The feasibility of team care for women seeking to plan a vaginal breech birth (OptiBreech 1) – an observational implementation feasibility study in preparation for a pilot trial. BMC Pilot & Feasibility Studies. 2022;In Review.) which has currently not yet been accepted for publication. Please remove this from your References and amend this to state in the body of your manuscript: (ie “Bewick et al. [Unpublished]”) as detailed online in our guide for authors
<http://journals.plos.org/plosone/s/submission-guidelines#loc-reference-style>

**We are pleased that this paper has now been published and have updated the reference accordingly.**

4. We note that the original protocol that you have uploaded as a Supporting Information file contains an institutional logo. As this logo is likely copyrighted, we ask that you please remove it from this file and upload an updated version upon resubmission.

**Changed and version without logos uploaded.**

5. Please include captions for your Supporting Information files at the end of your manuscript, and update any in-text citations to match accordingly. Please see our Supporting Information guidelines for more information: <http://journals.plos.org/plosone/s/supporting-information>.

**Done.**

6. Please review your reference list to ensure that it is complete and correct. If you have cited papers that have been retracted, please include the rationale for doing so in the manuscript text, or remove these references and replace them with relevant current references. Any changes to the reference list should be mentioned in the rebuttal letter that accompanies your revised manuscript. If you need to cite a retracted article, indicate the article’s retracted status in the References list and also include a citation and full reference for the retraction notice.

**This has been checked. The only changes are formatting and the full reference for the paper recently published. To the best of our knowledge, we have not cited any retracted articles.**

Additional Editor Comments:

Dear Authors,
the Reviewers are favorable to the publication of your manuscript after a minor revision.
Please incorporate or discuss the suggested changes and submit a revised version of your manuscript in order to achieve publication.
Best regards

Salvatore Andrea Mastrolia
Academic Editor

**Wonderful news! Thank you for your team’s careful review and guidance to improve this manuscript.**


Reviewers' comments:

Review Comments to the Author

Reviewer #1: The primary objectives of this pilot feasibility clinical trial were to evaluate recruitment, acceptance and attrition rates for women randomized to a ‘new care process’. A statistically significantly higher proportion of women planned a VBB when randomized to OptiBreach Care. The trial terminated prematurely.

Minor revisions:
1- A. Line 239: Indicate the statistical method used for the “two-sided test.”

**Thank you. We have now clarified that a chi-square test was used.**

B. Line 295: Indicate the statistical methods used for comparing proportions. For example, 0/33 compared to 8/24 with a p = 0.003.

**Thank you. This has been clarified with a detailed description. The denominator in the second proportion is 34 (consistent with original manuscript submission).**

Also remove the less than sign from the p-value.

**Removed.**

2- Table 4: State the type of summary statistic provided following the + signs.

**We have clarified in the table that this refers to weeks + days gestation.**

3- Lie 296: Indicate if the Agresti-Caffo CI is a 95% CI.

**95% added.**

4- Line 327: State the comparison group for women randomized to OptiBreech.

**Stated.**

5- Table 7: Define the abbreviation SD at its first occurrence.

**Defined in Table 6.**

6- Line 403: Clarify that the CI is associated with 23.5%.

**Done.**

**We hope this has satisfactorily addressed the statistical reviewer’s concerns. Many thanks again for your careful attention to detail, which has improved this manuscript.**
