# Supplementary material for: OptiBreech collaborative care versus standard care for women with a breech-presenting fetus at term: A pilot parallel group randomised trial to evaluate the feasibility of a randomised trial nested within a cohort
Source: PLoS One. 2023 Nov 15;18(11):e0294139. doi: 10.1371/journal.pone.0294139 (PMC10650999; doi:10.1371/journal.pone.0294139)
Supplement: Attachment — Submitted filename: Response to Reviewers R2.docx [file pone.0294139.s006.docx]

**Response to Reviewers**

Thank you for your further careful review of our manuscript and suggestions for improvement.

Please include the following items when submitting your revised manuscript:

- A rebuttal letter that responds to each point raised by the academic editor and reviewer(s). You should upload this letter as a separate file labeled 'Response to Reviewers'. Responses below.
- A marked-up copy of your manuscript that highlights changes made to the original version. You should upload this as a separate file labeled 'Revised Manuscript with Track Changes'. Uploaded.
- An unmarked version of your revised paper without tracked changes. You should upload this as a separate file labeled 'Manuscript'. Uploaded.

If you would like to make changes to your financial disclosure, please include your updated statement in your cover letter.

No changes to those already submitted.

Journal Requirements:

Please review your reference list to ensure that it is complete and correct. If you have cited papers that have been retracted, please include the rationale for doing so in the manuscript text, or remove these references and replace them with relevant current references. Any changes to the reference list should be mentioned in the rebuttal letter that accompanies your revised manuscript. If you need to cite a retracted article, indicate the article’s retracted status in the References list and also include a citation and full reference for the retraction notice.

Checked.

Additional Editor Comments:

I would like to suggest the author to address reviewer 1 comment "In the reply the author indicate that proportions were compared with the chi-square test. In addition, the chi-square test should be noted in the manuscript."

It is more correct to say we used a Fisher exact test, as one of the cells contained zero. This is now noted in the manuscript (line 234).

Reviewer's Responses to Questions

**Comments to the Author**

Reviewer #1: Minor revision:
In the reply the author indicate that proportions were compared with the chi-square test. In addition, the chi-square test should be noted in the manuscript.

It is more correct to say we used a Fisher exact test, as one of the cells contained zero. This is now noted in the manuscript (line 234).

Reviewer #2: I find this project to be truly intriguing, and I would like to express my appreciation to the authors for diligently addressing the previous comments. I recommend publishing this paper as it significantly contributes to the existing literature. However, I do have a question regarding the excessive use of the authors' own references, which appears to exceed 16 instances. I wonder if this is a deliberate strategy to increase citations to their previous work. Nevertheless, despite this concern, I maintain a favorable opinion of the paper and believe it offers valuable insights to the field.

This is a reasonable concern! However, the strategy is to demonstrate that this complex intervention is the result of over a decade’s worth of theoretical work and evaluation of its components. We have also become aware of some guidelines using our work, particularly the time interval recommendations in the Physiological Breech Birth Algorithm, without citation. We want to be clear about how the intervention has been developed and that we are not just testing ‘vaginal breech birth’ according to the individual provider’s preferences.

Two of the citations are for the protocol registration and the data repository. K Walker is no relation and not part of the current research team.

Nonetheless, we will take this to heart and write an article that dissects the complex intervention. Then we will be able to refer succinctly to one summary publication in the future.

Reviewer #3: I would like to thank and congratulate the authors for their interesting work, now the manuscript is fit or suitable for publication.

Thank you.

While revising your submission, please upload your figure files to the Preflight Analysis and Conversion Engine (PACE) digital diagnostic tool, [https://pacev2.apexcovantage.com/](https://eur03.safelinks.protection.outlook.com/?url=https%3A%2F%2Fpacev2.apexcovantage.com%2F&data=05%7C01%7Cshawn.walker%40kcl.ac.uk%7Cd557000ebd5c454c33f508dbcf941bff%7C8370cf1416f34c16b83c724071654356%7C0%7C0%7C638332007254736907%7CUnknown%7CTWFpbGZsb3d8eyJWIjoiMC4wLjAwMDAiLCJQIjoiV2luMzIiLCJBTiI6Ik1haWwiLCJXVCI6Mn0%3D%7C3000%7C%7C%7C&sdata=WHBodFuTopJA6OY1D0lfFEXyBmjsbUiuSKBPj2kwUTw%3D&reserved=0). PACE helps ensure that figures meet PLOS requirements. To use PACE, you must first register as a user. Registration is free. Then, login and navigate to the UPLOAD tab, where you will find detailed instructions on how to use the tool. If you encounter any issues or have any questions when using PACE, please email PLOS at [figures@plos.org](mailto:figures@plos.org). Please note that Supporting Information files do not need this step.

Done.
